# Allosteric Communication in Myosin V: From Small Conformational Changes to Large Directed Movements

**DOI:** 10.1371/journal.pcbi.1000129

**Published:** 2008-08-15

**Authors:** M. Cecchini, A. Houdusse, M. Karplus

**Affiliations:** 1 Laboratoire de Chimie Biophysique, Université Louis Pasteur (ISIS), Strasbourg, France; 2 Structural Motility, Institut Curie CNRS, UMR144, Paris, France; 3 Department of Chemistry and Chemical Biology, Harvard University, Cambridge, Massachusetts, United States of America; University of California San Francisco, United States of America

## Abstract

The rigor to post-rigor transition in myosin, a consequence of ATP binding, plays an essential role in the Lymn–Taylor functional cycle because it results in the dissociation of the actomyosin complex after the powerstroke. On the basis of the X-ray structures of myosin V, we have developed a new normal mode superposition model for the transition path between the two states. Rigid-body motions of the various subdomains and specific residues at the subdomain interfaces are key elements in the transition. The allosteric communication between the nucleotide binding site and the U50/L50 cleft is shown to result from local changes due to ATP binding, which induce large amplitude motions that are encoded in the structure of the protein. The triggering event is the change in the interaction of switch I and the P-loop, which is stabilized by ATP binding. The motion of switch I, which is a relatively rigid element of the U50 subdomain, leads directly to a partial opening of the U50/L50 cleft; the latter is expected to weaken the binding of myosin to actin. The calculated transition path demonstrates the nature of the subdomain coupling and offers an explanation for the mutual exclusion of ATP and actin binding. The mechanism of the uncoupling of the converter from the motor head, an essential part of the transition, is elucidated. The origin of the partial untwisting of the central *β*-sheet in the rigor to post-rigor transition is described.

## Author Summary

Myosins are molecular motor proteins that interact with actin filaments to perform a wide range of cellular functions. They use the universal energy storage molecule adenosine triphosphate (ATP). The functional cycle involves myosin binding to actin, a “powerstroke” leading to directed movement, and myosin release in preparation for the next step. A fundamental question concerns the mechanism by which the local structural changes due to ATP binding, hydrolysis, and products release can generate the large myosin changes of conformation required for this cycle. Here, we focus on the rigor to post-rigor transition of myosin V, which results in the release of myosin from actin. Starting from the X-ray structures of the two states, we have used the optimal superposition of normal modes to determine the transition path. The path shows the allosteric mechanism by which ATP binding leads to the opening of the U50/L50 cleft, the essential step in the unbinding of myosin from actin. More generally, the new normal-mode superposition model can be useful for describing large-amplitude conformational transitions encoded in protein structures by evolution.

## Introduction

Motion is one of the hallmarks of life. Myosins are molecular motor proteins that use ATP to power interactions with actin filaments, so as to generate force and directed movement. A variety of different myosins are found in eukaryotic cells from yeast to man [1]. They perform a wide range of essential functions, including transport of different particles (e.g., secretory vesicles), signal transduction, cell adhesion, endocytosis, muscle contraction, and cell mobility, amongst others. The class V myosins are of special interest because they act individually to carry out their function, which is the transport of vesicles inside the cell. Myosin V is composed of two catalytic domains (called “motor” domains), which can bind to actin. They each have long “lever arms”, which are joined to a coiled-coil region that ends in a globular domain for binding the cellular cargo; each lever arm consists of six IQ motifs to which light chains or calmodulin domains bind. Recently, it has been shown that myosin V moves along F-actin by a processive “hand-over-hand” mechanism with steps approximately 36 nm in length [2],[3] although the details of the stepping are still under investigation [4],[5]. Thus, myosin V poses in the clearest fashion the question of how the relatively small conformational changes occurring in the catalytic domains can result in very large displacements of the molecule as a whole. The most commonly accepted mechanism is the “swinging lever arm hypothesis” [6],[7]. This model proposes that small conformational changes in the motor domain are coupled to and amplified by the lever arm, whose position is controlled by the rotation of the converter subdomain. Given such coupling, it is likely that the induced motion is diffusive and corresponds to transitions between different conformational states which are stabilized by the nature of the ligand; i.e., no ligand; ATP; ADP·P_i_; ADP. This implies that the motor domain cycles through well-defined, though fluctuating, structural states which differ in their nucleotide and actin binding affinities. Such a description is analogous to the binding change mechanism for F_1_-ATPase [8],[9]. Myosin V binds strongly to actin in the absence of ATP and apparently hydrolyzes ATP in a state that has a relatively weak affinity for actin. Binding to actin accelerates the release of P_i_ (H_2_PO_4_^−^), which promotes the transition from the weak to the strong actin-binding myosin states [10]. Strain is thought to be introduced when both myosin heads bind to the actin filament, and their conformations are altered. As a result, the release of ADP from the trailing head is slightly accelerated and that from the leading head is significantly slowed down [11],[12].

**Figure 1:**
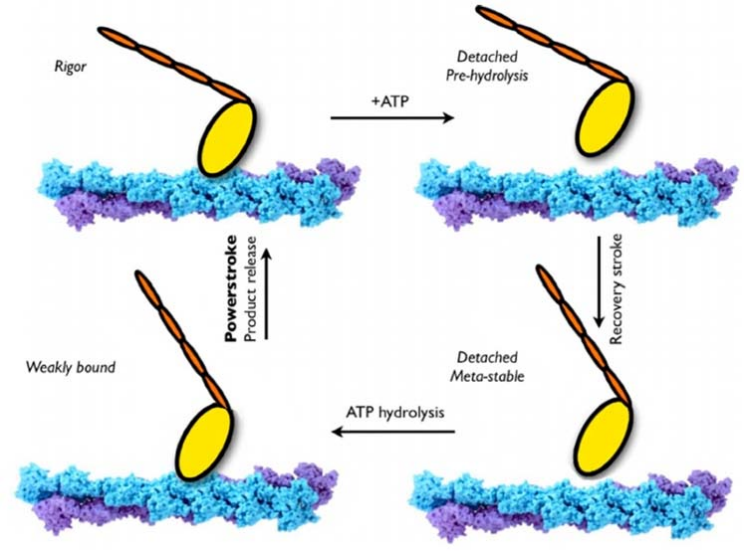
The Lymn–Taylor functional cycle of the actomyosin complex [6],[13] (adapted from Yu et al. [19] to indicate the motion of the lever arm appropriate for myosin V). Only a myosin monomer is shown for simplicity. Binding of ATP to the actomyosin complex (the rigor state) leads to rapid dissociation of myosin from actin without immediate hydrolysis of ATP. Coupled with a major structural change in the orientation of the lever arm (“recovery stroke”), ATP hydrolysis proceeds and the motor domain weakly rebinds to actin. Following the release of P_i_, the motor domain undergoes the “powerstroke” during which the lever arm moves back to the rigor state and the motor domain becomes strongly bound to actin. Dissociation of ADP leads the system back to the rigor state.

As formulated by Lymn and Taylor [13], the myosin V cycle can be regarded as consisting of two parts: the states corresponding to free myosin (i.e., myosin not bound to actin) and the states of the actomyosin complex (Figure 1). At the present time, no high resolution structures of the latter are available, although there is evidence from cryo-electron microscopy that one of the structures recently obtained for myosin V with no nucleotide bound [14] (referred to as the rigor-like conformation) corresponds to the strongly bound actomyosin complex (the so-called rigor state) [15]. There is also a structure for the post-rigor state of myosin V [16], as well as for myosin II [17], formed when ATP binds to actomyosin and myosin dissociates from actin. The post-rigor state is in equilibrium with the pre-powerstroke state, which is stabilized by P_i_ and ADP in the active site after ATP hydrolysis [18],[19]. Myosin then binds to actin with the lever arm in a position corresponding to the beginning of the powerstroke, i.e., the force generation phase of the cycle. Release of the hydrolysis products, P_i_ and ADP, and the powerstroke follow; the specific structures of actomyosin involved at this stage are not known. Rebinding of ATP causes myosin to dissociate from actin and a new cycle begins.

Some insights concerning how myosin functions have been obtained by comparing sets of structures that are thought to represent various stages in the cycle. These analyses are based on the implicit assumption that an essentially linear interpolation path can describe the change from one conformation to another. To obtain a more complete understanding of the actual motions, it is necessary to complement the structural data by simulations. For the post-rigor to pre-powerstroke transition considerable information has been obtained by use of a variety of simulation methods [20],[21]. Here, we present the first study of the rigor to post-rigor transition by using myosin V structures and employing normal mode (NM) analysis [22]–[24], a well established method for studying the conformational changes of biomolecules [25]–[29]. For the post-rigor to pre-powerstroke transition in myosin II [30]–[33], several normal-mode based studies have been published. In this report, a Block Normal Mode (BNM) approach [34],[35] is used to explore the relationship between structure, ligation and function. The availability of the high resolution rigor-like state for myosin V [14] offers the first opportunity to study the dynamics of the so called “cleft-opening” motion, which is one of the essential elements in unbinding from actin. A structure for a rigor-like state of scallop myosin II with a closed cleft [36] was published after the present analysis was essentially complete; it supports the significance of the myosin V structure.

**Figure 2:**
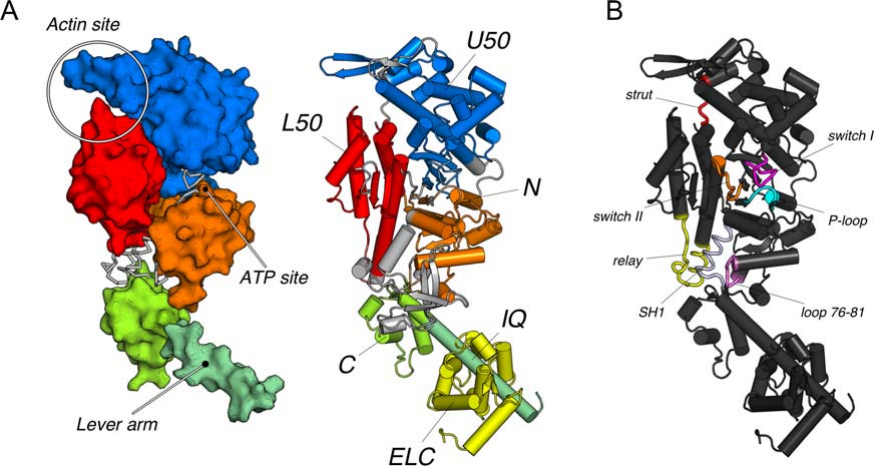
The myosin motor domain presented in the rigor-like conformation. (A) The motor subdomains and its functional sites. The nearly rigid motor subdomains are shown in space-filling models (on the left) and cartoons (on the right). The N-terminal (N), the upper 50 kDa (U50), the lower 50 kDa (L50), the converter (C), the first IQ motif (IQ), and the essential light chain (ELC) are colored in orange, blue, red, lime, pale green, and yellow, respectively. In the space-filling representation the location of the myosin functional sites is indicated: the actin-binding site at the interface of the U50 and L50 subdomains; the nucleotide-binding site at the interface of the N and U50 subdomains; and the beginning of the lever arm, whose position is controlled by the rotation of the converter. (B) The subdomain connectors in the myosin motor domain. The various connectors are color-coded as follows: the P-loop, switch I, switch II, the strut, the relay, helix SH1, and loop 76–81 are cyan, magenta, orange, red, yellow, slate, and violet. The P-loop, switch I and switch II contribute to the formation of the active site involved in nucleotide binding and hydrolysis at the interface of the N, U50, and L50 subdomains; the strut joins U50 and L50 in the upper part of the U50/L50 cleft; the relay group connects L50 to C; helix SH1 and loop 76–81 connect the converter to the N-terminal subdomain. In the text, the terms “upward” and “downward” are used to indicate motion in the direction of the top and bottom of the figure.

Comparison of the rigor-like unliganded structure with the ATP-bound post-rigor structure has provided information concerning the structural changes involved [16]. In the present paper, we elucidate the nature of the coupling among the characterized subdomains and connectors (see Figure 2) that results in the rigor to post-rigor transition. Analysis of forty of the low-frequency modes makes possible a determination of the motions that are likely to be important. Using a method that determines a pathway from the rigor-like to the post-rigor state based on the superposition of the normal modes we are able to obtain insights into the mechanism of allosteric communication in myosin V. The next section describes the materials and methods. The results are presented in the third section. A discussion of the significance of the results is given in the ending section.

## Materials and Methods

### Structures

The proteins were modeled with the polar-hydrogen potential function [37] and solvation effects were approximated by an effective solvent model, EEF1 [38], which contains screened electrostatic interactions and a Gaussian term to represent full hydrophobic interactions. The initial coordinates of the myosin molecule in the rigor-like and post-rigor functional states were obtained from the PDB (PDB entries 1OE9 and 1W7J, respectively). The rigor-like structure is a nucleotide-free myosin conformation, while the post-rigor structure represents an ATP-bound state. The RMS difference between the rigor-like and post-rigor structures is 5.4 Å (5.2) for X-ray and 5.3 Å (5.2) after energy minimization; the all heavy-atom result is given first and the C_α_ result in parentheses. Details concerning the model and the preparation of the structures for the normal mode calculations are given in Text S1. The structural definition of the various subdomains, secondary-structure elements, and linkers of myosin V is given in Table S1 and Table S2.

### Normal Mode Calculation

Normal mode analysis was performed on both functional states of myosin V by using the block normal mode (BNM) method [30],[35] as implemented in the program CHARMM [39]. In the BNM approach, the molecule is partitioned into moving units (blocks, here each unit contains one residue) and the Hessian matrix is projected onto the subspace spanned by the translation and rotation vectors of the blocks. Even though the internal degrees of freedom of the moving units are frozen in the BNM description, which limit the side-chain motions, previous studies have shown that the results are appropriate for the investigation of the modes of primary interest for conformational changes [34],[35],[30]. For details concerning the normal mode calculation, analysis and inherent limitations see Text S2.

### Overlap Coefficients

To compare individual modes of the rigor-like and post-rigor conformations, overlaps between pairs of eigenvectors belonging to the different functional states were computed. The rigor-like and post-rigor conformations were first aligned by fitting all C_α_ atoms which were not missing in the original X-ray structures (i.e., 837 centers out of the total of 908 included in the normal modes). The overlap coefficient, *C_ij_*, between modes *i* and *j* of structures *α* and *β*, respectively, is defined as the dot product of the corresponding eigenvectors **L** *_i_* and **L** *_j_*

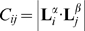
(1)


Large overlaps (i.e., *C_ij_* values close to one) indicate that the eigenvectors point in the same direction, so that they describe similar harmonic oscillations.

### Involvement Coefficients

To determine the mode contribution to the rigor-like/post-rigor transition, individual and cumulative involvement coefficients [25]–[27],[30] were computed. A transition pathway is determined by linearly interpolating the transition end-points (i.e., the initial, 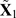, and final, 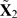, structures of a conformational transition) upon optimal superposition of all the C_α_ atoms. The resulting “displacement vector” is expanded as a linear combination of normal modes of the initial state. The projection of the normalized displacement vector on the *k*th normal mode vector, **L** *_k_*, is computed as
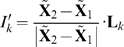
(2)


The corresponding involvement coefficient is defined as 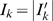, which describes the degree of involvement of the *k*th mode in the conformational transition. Thus, the individual involvement coefficients indicate in a semi-quantitative way which collective motions are important for a given conformational change. A complementary quantity that indicates the weight of a set of normal modes along the transition is the cumulative involvement coefficient 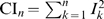. In the present case, as we shall see, a small number of low-frequency modes (40 out of a total of 5,448 in the residue-based BNM analysis) appear to give a highly accurate description of the motions along the rigor-like/post-rigor transition. “Specialized” involvement-coefficient analyses are used to identify the mode contributions to the structural transition of specific elements. In such analyses, suitably defined portion of the molecule are considered separately and a “specialized” displacement vector is determined by zeroing the elements of the original vector that correspond to atoms outside the region of interest.

### Dynamic Domain Analysis

The program DynDom [28] (version 1.5) was used to analyze the interdomain conformational change described by the low-frequency modes and identify the dynamic domains and hinge regions.

### Generation of Transition Pathway

The linear combination of a subset of normal modes, weighted by their involvement coefficients is optimal for describing a given conformational change [26]; i.e., it gives the smallest RMSD relative to the target. In this approach, the involvement coefficients are used to superpose a subset of *M* modes, starting with the first *M* lowest-frequency modes after removing the translational and rotational modes. The evolution vector of the structure based on the involvement coefficients is given by
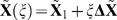
(3)where 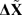, the optimal superposition of the considered modes, is given by
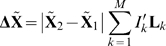
(4)


The evolution coefficient, *ξ*, is the fractional evolution of the structure along the transition path; *ξ* varies from 0 to 1. The coefficients, 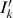, of the linear combination are the “signed” involvement coefficients, as defined by Equation 2. At the beginning of the evolution (*ξ* = 0) 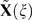 corresponds to the starting state; at the end (*ξ* = 1) 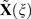 represents the molecular structure corresponding to the minimal RMSD from the final state that can be obtained by the set of *M* normal modes; it is hereafter referred to as the normal mode superposition model (NMSM) conformation 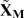. We use 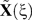 to study the evolution from the initial (rigor-like) state 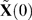, to the best approximation to the final (post-rigor) state, 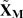. The NMSM evolution is used to obtain insights into the allosteric communication in the myosin head that leads from the binding of ATP in the rigor-like structure to the post-rigor structure (see Results).

## Results

In this section, we first determine the contributions of the low-frequency internal modes (it turns out that only 40 modes out of 5,448 are needed) to the global changes and subdomain displacements involved in the transition between the rigor-like and post-rigor states. We then combine the modes into a vector that gives a description of the transition and provides an understanding of the mechanism by which the local structural changes induced by ligand binding are coupled to achieve an overall transition from the rigor to the post-rigor state.

### Normal Mode Contributions to Flexibility and the Transition

**Figure 3:**
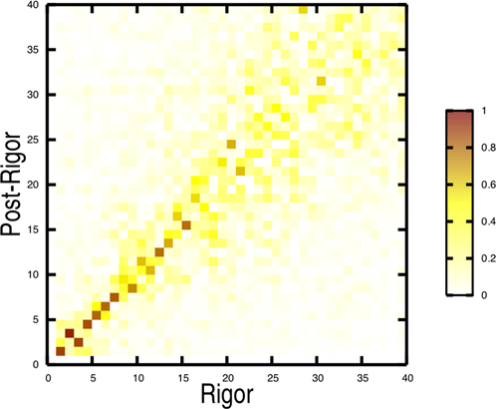
Rigor-like/post-rigor normal mode overlaps (see text). Dark colors indicate large overlaps (values close to unity) and correspond to strongly correlated motions.

The lowest-frequency internal normal modes (i.e., the overall translational and rotational modes have been removed) are very similar in the rigor-like and post-rigor states (see Figure 3). As shown by the overlap map, modes 1–7 are strongly correlated (overlaps >0.8), and modes 9–15, with the exception of mode 14, plus modes 20 and 21 are significantly correlated (overlaps >0.6); see Table S3. Higher-frequency modes (other than mode 30) show considerably less correlation. The 21 lowest-frequency modes have a frequency range of only 3.5 cm^−1^ (see Table S3) and a number of them are interchanged in the two structures: 2–3, 8–9, 10–11, and 14–16. The high correlation between the low-frequency modes of the two functional states, which represent distinct myosin conformations (C_α_-RMSD of 5.2 Å) is striking. The intrinsic “robustness” manifested by these modes suggests that they are likely to be functional [40] and, therefore, of direct interest.

### Involvement Coefficient Analyses

**Figure 4:**
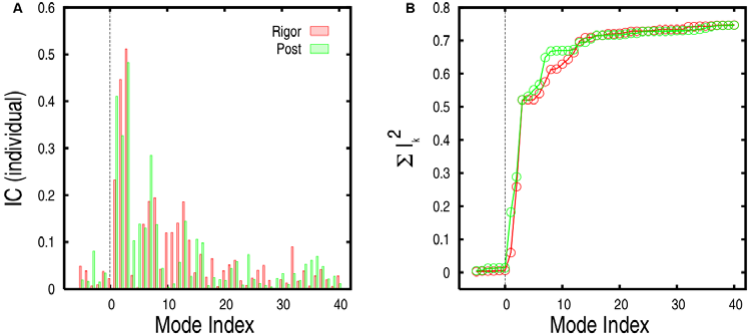
Involvement-coefficient analysis of the rigor-like/post-rigor conformational transition (see text). Individual (A) and cumulative (B) involvement coefficients are shown for the “forward” (from rigor-like to post-rigor) and “backward” (from post-rigor to rigor-like) transitions in red and green, respectively. Negative indices correspond to pure translational and rotational modes.

The relevance of the low-frequency modes of both the rigor-like and post-rigor states to the conformational transition can be quantified by their involvement coefficients (ICs); see Materials and Methods. Individual and cumulative involvement coefficients computed for the two states are reported in Figure 4. The first internal 15 (40) modes are sufficient to describe about 71% (75%) of the transition starting with either state, as measured by the cumulative ICs. Modes 1–3, 4–15, and 16–40 contribute 52%, 19%, and 4% of the conformational change, respectively.

The rigor-like and post-rigor states activate their normal modes somewhat differently in making the conformational transition. The first three modes, which show the largest involvement coefficients, behave similarly in the two structures. However, the activation of modes 4–15, which leads to a cumulative IC of 71% is specific to each conformation (see Figure 4B). In rigor-like, each normal mode brings a small contribution to the global change that results in a continuous increase of the cumulative curve; in post-rigor, the mode contribution is less homogeneous (see modes 7 and 13, in particular) and results in a more step-wise cumulative profile. An involvement-coefficient analysis performed on the “backward” (post-rigor to rigor-like) transition using a post-rigor structure without the nucleotide showed no changes in both the direction of the low-frequency eigenvectors and the mode activation pattern to the rigor-like state (see Text S3). This demonstrates that the primary effect of ATP is to stabilize the post-rigor structure, rather than to alter the intrinsic myosin flexibility.

**Figure 5:**
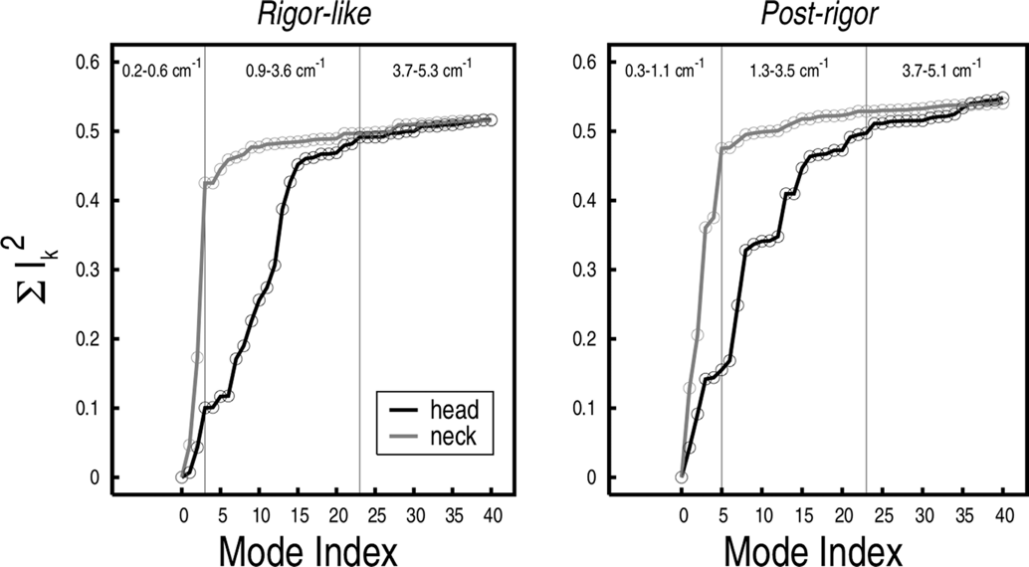
Involvement coefficients specialized for the rigor-like/post-rigor transition of the head domain and the neck region. The frequency range for the specialized modes is indicated.

To identify the structural contributions of the individual modes to the observed transition, a “specialized” involvement-coefficient analysis was carried out for both functional states (see Materials and Methods). As a first step, we focus on the two largest functional regions of the myosin molecule present in the crystal structure: the entire motor domain responsible for ATP hydrolysis (named the “head”), which structurally corresponds to the N, U50, and L50 subdomains, and the region connecting the motor domain to the lever arm (named the “neck”), which includes the converter, the first IQ motif and the essential light-chain (see Figure 2). The specialized involvement coefficients for the 40 lowest-frequency modes determined for these two portions of the molecule are reported in Table 1. The first three modes show very large involvement coefficients for the conformational transition of the neck region. The activation of these modes plus mode 5 in post-rigor, is sufficient to describe the change in the converter/lever-arm region, whereas slightly higher-frequency modes (i.e., modes 7–15) are required for the internal rearrangements of the motor domain (see Figure 5); modes 16–40 contribute relatively little to the overall transition (4% of the transition) but they are included because they are important for a better description of the more local changes.

**Table 1:** Rigor-like and post-rigor involvement coefficients for the conformational transition of the entire molecule (head plus neck), the head domain (N, U50, and L50; aa 61–699) and the neck region (C, IQ, and ELC; aa 700–946).

Mode #	Head+Neck		Head		Neck		Mode #	Head+Neck		Head		Neck	
	Rigor	Post	Rigor	Post	Rigor	Post		Rigor	Post	Rigor	Post	Rigor	Post
1	**0.23**	**0.41**	0.08	**0.21**	**0.22**	**0.36**	21	0.05	0.04	0.10	0.14	0.08	0.05
2	**0.45**	**0.33**	**0.19**	**0.22**	**0.36**	**0.28**	22	0.06	0.06	0.05	0.06	0.01	0.06
3	**0.51**	**0.48**	**0.24**	**0.22**	**0.50**	**0.39**	23	0.02	0.01	0.10	0.05	0.01	0.00
4	0.03	0.10	0.03	0.05	0.01	0.12	24	0.01	0.07	0.00	0.12	0.01	0.00
5	0.00	**0.14**	0.13	0.11	0.14	**0.32**	25	0.02	0.02	0.02	0.01	0.04	0.03
6	**0.14**	**0.13**	0.03	0.11	0.12	0.03	26	0.04	0.01	0.01	0.05	0.01	0.01
7	**0.19**	**0.29**	**0.23**	**0.28**	0.06	0.10	27	0.05	0.01	0.07	0.02	0.03	0.02
8	**0.19**	**0.14**	0.14	**0.28**	0.06	0.10	28	0.02	0.00	0.01	0.02	0.10	0.00
9	0.04	0.04	**0.19**	0.09	0.10	0.05	29	0.00	0.02	0.05	0.00	0.01	0.04
10	**0.12**	0.00	**0.17**	0.07	0.01	0.04	30	0.02	0.03	0.01	0.02	0.03	0.02
11	**0.12**	0.01	0.13	0.03	0.06	0.03	31	0.01	0.01	0.08	0.06	0.02	0.04
12	**0.14**	0.06	**0.18**	0.08	0.02	0.03	32	0.09	0.03	0.02	0.04	0.01	0.04
13	**0.19**	**0.14**	**0.29**	**0.25**	0.04	0.08	33	0.02	0.02	0.00	0.03	0.05	0.04
14	**0.10**	0.03	**0.20**	0.01	0.02	0.07	34	0.04	0.05	0.05	0.05	0.01	0.01
15	0.03	**0.11**	**0.16**	**0.19**	0.03	0.07	35	0.01	0.06	0.00	0.10	0.04	0.00
16	0.07	**0.10**	0.09	0.13	0.03	0.00	36	0.03	0.07	0.04	0.08	0.01	0.02
17	0.03	0.01	0.04	0.05	0.04	0.06	37	0.04	0.05	0.05	0.03	0.02	0.03
18	0.06	0.02	0.07	0.03	0.03	0.03	38	0.02	0.02	0.03	0.05	0.00	0.01
19	0.00	0.02	0.01	0.07	0.02	0.01	39	0.01	0.03	0.05	0.02	0.02	0.03
20	0.04	0.02	0.04	0.01	0.03	0.03	40	0.03	0.01	0.01	0.06	0.00	0.00

The involvement coefficients are given for both “forward” (from rigor-like to post-rigor) and “backward” (from post-rigor to rigor-like) directions. Modes contributing more than 0.10 and 0.15 to the structural rearrangement of the entire molecule and its domains, respectively, are shown in bold.

To determine the internal rearrangements of the motor domain that involve the subdomain displacements, another specialized involvement-coefficient analysis was carried out. Upon optimal superposition of all C_α_-atoms belonging to the motor domain (aa 61–762), the rigor-like/post-rigor “displacement vector” was decomposed into five subvectors, one for the entire motor domain and four corresponding to the individual subdomains (i.e., N, U50, L50, and C). The results of the analysis are reported in Table S5. Several modes in the medium-frequency range, from mode 7 to mode 24, have involvement coefficients larger than 0.15 and contribute significantly to the conformational transition of the motor: modes 8, 9, 10, 11, 12, 13, 14, 15, 18, and 21 in rigor-like, and modes 7, 8, 13, 15, 21, and 24 in post-rigor. To investigate the correlation between the various subdomain motions, pairs of specialized involvement coefficients that are relatively large in magnitude and of the same sign were identified. Pairs of involvement coefficients of the same sign for a given mode indicate a correlated motion of the corresponding subdomains, while pairs of opposite sign indicate an anticorrelated motion. As reported in Table 2, modes 13, 10, 8 and 21, 14 and 12, 9, and 11 in rigor-like describe the relevant coupling between the N/U50/L50, U50/L50, L50/C, N/L50, N/U50, and U50/C subdomains, respectively. Similarly, modes 8 and 13, 7, and 15 in post-rigor are involved in the coupled rearrangements of the N/U50/L50, N/U50, and U50/C subdomains, respectively. The fact that a different number of rigor-like and post-rigor modes are primarily involved in the specific rearrangements of the motor domain confirms that there is a slight change in flexibility as a consequence of the structural changes due to nucleotide binding (see above).

**Table 2:** Subdomain coupling on the most-involved low-frequency modes.

Functional state	Mode #	Motor	N	U50	L50	C	Subdomain coupling
**Rigor-like**	13	**0.31**	**0.29**	**0.13**	**0.14**	0.01	N/U50/L50
	10	**0.23**	0.05	**0.23**	**0.17**	−0.08	U50/L50
	15	**0.21**	−0.07	**−0.24**	0.02	−0.09	U50
	8	**0.20**	−0.06	−0.06	**−0.16**	**−0.15**	L50/C
	14	**0.20**	**−0.12**	0.01	**−0.18**	−0.10	N/L50
	12	**0.19**	**0.14**	0.06	**0.15**	−0.02	N/L50
	9	**0.18**	**0.12**	**0.17**	0.08	−0.02	N/U50
	21	**0.17**	−0.04	0.08	**0.15**	**0.19**	L50/C
	18	**0.15**	**−0.20**	−0.07	**0.12**	−0.09	N
	11	**0.15**	−0.06	**−0.13**	0.04	**−0.17**	U50/C
	7	0.12	**−0.12**	**−0.13**	0.02	0.07	N/U50
	16	0.11	**−0.16**	**0.14**	**0.12**	0.08	U50/L50
	17	0.09	**−0.20**	0.02	**0.14**	**−0.14**	N/C
**Post-rigor**	8	**0.35**	**0.15**	**0.21**	**0.25**	0.06	N/U50/L50
	13	**0.33**	**−0.19**	**−0.20**	**−0.20**	0.02	N/U50/L50
	7	**0.28**	**0.13**	**0.21**	0.08	0.04	N/U50
	15	**0.23**	0.05	**0.25**	−0.04	**0.16**	U50/C
	24	**0.17**	**−0.17**	−0.03	−0.03	−0.09	N
	21	**0.15**	**0.15**	−0.11	**−0.29**	−0.09	L50
	16	0.11	**0.12**	−0.10	0.11	**0.12**	N/C

Rigor-like and post-rigor involvement coefficients relative to the motor domain and the individual subdomains sorted by their contribution to the transition of the motor domain (aa 61–762). The coupling between the individual subdomains along the most involved normal modes is given, as deduced by the analysis of the signed involvement coefficients.

### Intrinsic Flexibility of the Myosin Neck

**Figure 6:**
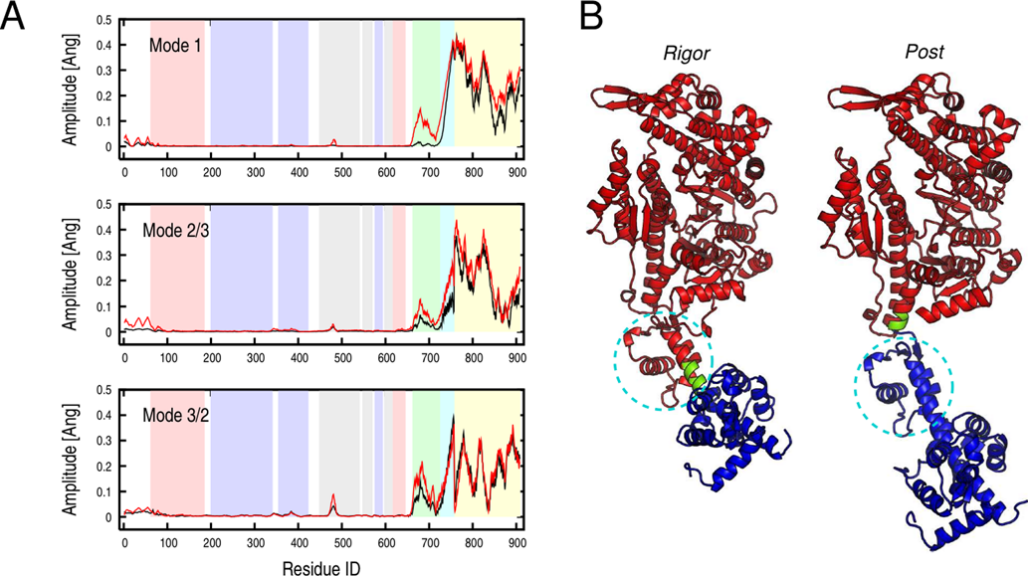
Rigor-like and post-rigor lowest-frequency modes that are essentially independent of the motor head. (A) Amplitude of the C_α_ fluctuations along the sequence computed upon optimal superposition of the head domain (aa 61–699) for the three lowest-frequency modes. Black and red profiles correspond to the rigor-like and post-rigor states, respectively. In the background, pink, light blue, grey, light green, cyan, and yellow indicate the boundaries of the N, U50, L50, C, IQ, and ELC subdomains. Rigor-like and post-rigor fluctuations show a difference in the conformational freedom of the converter in the two states (light green region). (B) DynDom analysis of the rigor-like and post-rigor lowest-frequency modes. In both cases two dynamic domains corresponding to the head domain (red) and the neck region (blue) are identified. The analysis indicates that the converter subdomain (shown surrounded by a dashed circle) belongs to the head domain in the rigor-like state and to the neck region in the post-rigor state.

The lowest-frequency modes of myosin V in both rigor-like and post-rigor states (i.e., modes 1–3 with frequencies ranging from 0.22 to 0.58 cm^−1^, see Table S4) dominate the motion of the converter/lever region (i.e., the “neck”) with respect to the myosin head. The motor domain essentially moves as a rigid unit. The amplitudes of the C_α_ fluctuations computed upon optimal superposition of the head (aa 61–699) clearly show that only very small fluctuations are present in the N, U50 and L50 subdomains with the exception of the relay group (aa 467–493) that is coupled to the converter (see Figure 6A). Mode 1 shows a large swing of the lever arm in a direction that is essentially perpendicular to the observed conformational transition, while modes 2 and 3 involve the twisting of the lever arm in a clockwise and counterclockwise direction, respectively. In the latter two, the twisting is coupled to a short-amplitude swinging motion; i.e., a rotation of the lever arm as a whole. In mode 3 for rigor-like, which corresponds to mode 2 in post-rigor (see above), the displacement corresponds to the direction of the observed conformational transition, which results in the large involvement coefficients (see Table 1). The collective motions described by these modes are similar in both states and essentially split the molecule in two dynamic domains, i.e., the head domain and the neck region, as determined by DynDom (red and blue regions in Figure 6B). An important point is that the converter belongs to the head in the rigor-like state and to the neck in the post-rigor state (see Figure 6B); the molecular mechanism leading to the uncoupling of the converter from the motor head is described below. The DynDom analysis of modes 1–3, indicates that the hinge is located at residues 763–769 in rigor-like (i.e., at the pliant region located at the beginning of the lever arm [41]), and at residues 696–697 in post-rigor (i.e., at the junction between the SH1 helix and the converter); see Figure 6B, in green. Due to the different location of the hinges, the converter subdomain tends to be more independent of the head in post-rigor than in rigor-like. This is likely to be important for making possible the transition to the pre-powerstroke state; kinetically the post-rigor and pre-powerstroke states are in equilibrium with ATP bound [18].

**Figure 7:**
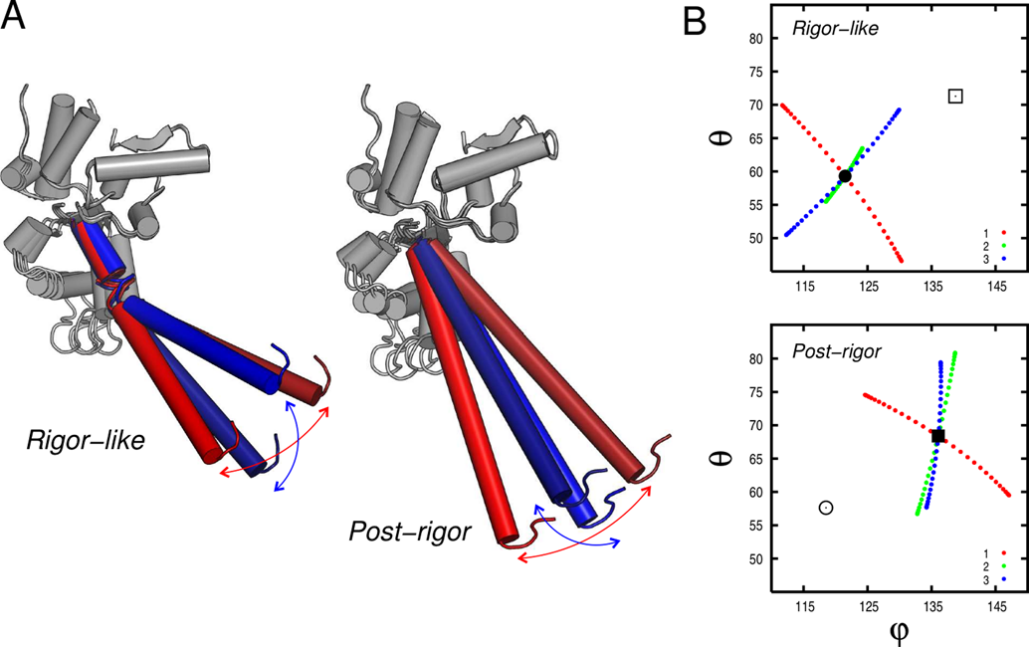
Lever-arm motion encoded in the lowest-frequency modes in the rigor-like and post-rigor states of myosin V. (A) Pictorial representation of the lever oscillation along modes 1 (red) and 3 (blue) in the rigor-like and post-rigor states. (B) Lever-arm motion reported in spherical coordinates along the lowest-frequency modes. The spherical coordinates *ϕ* and *θ*, which correspond to the zenith and the azimuth angle, respectively, were determined by fitting the coordinates of the lever-arm backbone atoms (aa 754–792) upon optimal superposition of the N, U50, and L50 subdomains to the equilibrium structure. The circle and square correspond to the orientation of the lever arm in the rigor-like and post-rigor conformations, respectively. Mode 1 and 3 are essentially perpendicular in both functional states.

In Figure 7, the direction of the lever-arm motion as described by the first three lowest-frequency modes is shown. Modes 1 and 3 are essentially perpendicular in both states, with mode 3 in the direction of the transition in rigor-like, in accord with the involvement coefficient (see Table 1).

### Allosteric Communication in the Myosin Head

**Figure 8:**
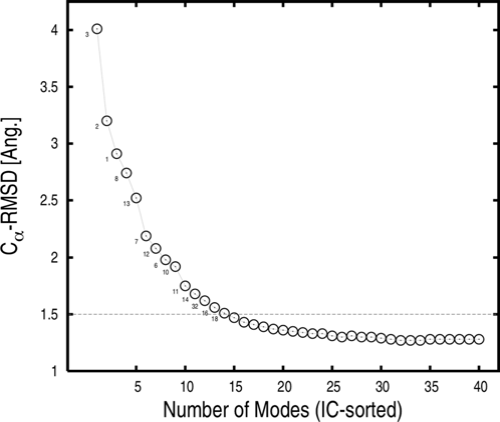
C_α_-RMS deviation of the NMSM post-rigor conformation from the “target” (i.e., the X-ray post-rigor conformation after energy minimization) as a function of the number of low-frequency rigor modes included in the optimal superposition. The rigor modes were first sorted according to their rigor-like/post-rigor involvement coefficients and then combined as described in Materials and Methods. Only 14 modes are sufficient to obtain a NMSM conformation that is at 1.5 Å RMSD from the target structure. The mode indexes of these 14 highly involved modes are indicated.

An essential element in a complete description of the transition from the rigor to the post-rigor state in the myosin cycle is the elucidation of the mechanism by which nucleotide binding is coupled to the opening of the U50/L50 cleft. For this purpose, we computed the optimal superposition of the low-frequency modes of the rigor-like state to generate the transition vector 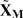 from the rigor-like state toward the post-rigor state; see Equations 3 and 4 in Materials and Methods. The superposition of the first 40 low-frequency modes based on the signed involvement coefficients produces a myosin conformation with a C_α_-RMSD of only 1.28 Å from the post-rigor conformation; we refer to the resulting conformation as the Normal-Mode Superposition Model (NMSM) conformation. This value is to be compared with the C_α_-RMSD of 5.2 Å for the entire molecule between the rigor-like and post-rigor energy-minimized conformations. The choice of the number of modes (40 modes) was based on the involvement coefficients; 40 modes represents only the 0.7% of the total (5,454 modes) in the BNM approximation. The analysis (Table 1) suggests that the use of 40 modes is sufficient to include both the large-scale motions and a significant fraction of the local rearrangements, such as those involving the switches in the ATP-binding site. Of these 40 modes, only 14 modes are required to describe the overall rigor-like/post-rigor conformational transition and lead to an RMSD of 1.5 Å (see Figure 8). Even though 40 modes give a detailed representation of the transition, still higher-frequency modes would have to be included to obtain a complete description of the local rearrangements, such that of the P-loop.

As a first step in analyzing the transition, we consider the rigor-like/post-rigor structural change as a combination of rigid-body subdomain movements. After optimal superposition of the C_α_ atoms of the rigor-like and the NMSM post-rigor conformation, the intermolecular distances between the centers of mass of the corresponding subdomains (N, U50, L50, C, and IQ/LC) in the two structures are equal to 4.2, 3.2, 0.9, 3.7, and 6.6 Å, respectively. These values are in good agreement with the corresponding distances in the X-ray conformations; they are 4.1, 2.9, 1.3, 3.2, and 7.2 Å, respectively. Large translational displacements are observed for the N, U50, C, and IQ/LC subdomains, while there is only a small translation of L50 (see Table 3, last column). N and U50 move upwards with respect to L50, while the converter and the lever arm move in the opposite direction, as shown by the increase of its distance from N, U50, and L50 (see Figure S1). The motion of U50 relative to L50 results in the partial opening of the U50/L50 cleft; i.e., both the N/U50 and N/L50 center-of-mass distances are essentially preserved, while U50 and L50 move away from each other by 1.9 Å (see Table S6). At the same time, the downward movement of the converter, which is followed by L50, weakens the coupling between the neck region and the head domain; i.e., both N/C and U50/C distance changes are almost twice as large as L50/C (see Table S6).

**Table 3:** Rigid-body description of the NMSM transition in terms of subdomain screw-axis transformations.

Subdomain	Axis	Vector vˆ			Vector ṽ _c_			*ϕ*	*d*	com
**N**	**nˆ**	−0.84	0.25	0.48	−2.28	−4.42	50.60	13.7	1.4	4.2
**U50**	**û**	−0.32	0.68	−0.66	−2.80	−10.70	70.60	10.4	2.0	3.2
**L50**	**lˆ**	−0.33	0.59	0.74	−0.95	−12.90	48.80	10.9	0.0	0.9
**C**	**ĉ**	0.34	0.29	−0.89	−15.57	10.40	13.50	12.4	2.9	3.7
**IQ/LC**	**qˆ**	0.74	−0.60	−0.31	−1.70	2.74	−1.54	14.4	0.3	6.6

Vectors **vˆ** and 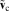 correspond to the position and orientation of the screw axis, scalars *ϕ* and *d* to the rotation angle and the translational shift involved in the screw-axis transformation, respectively. The screw axes for individual subdomains were determined as described in Text S4. The last column reports the intermolecular distances between the centers of mass (**com**) of the corresponding subdomains of the rigor-like and the NMSM conformation.

**Figure 9:**
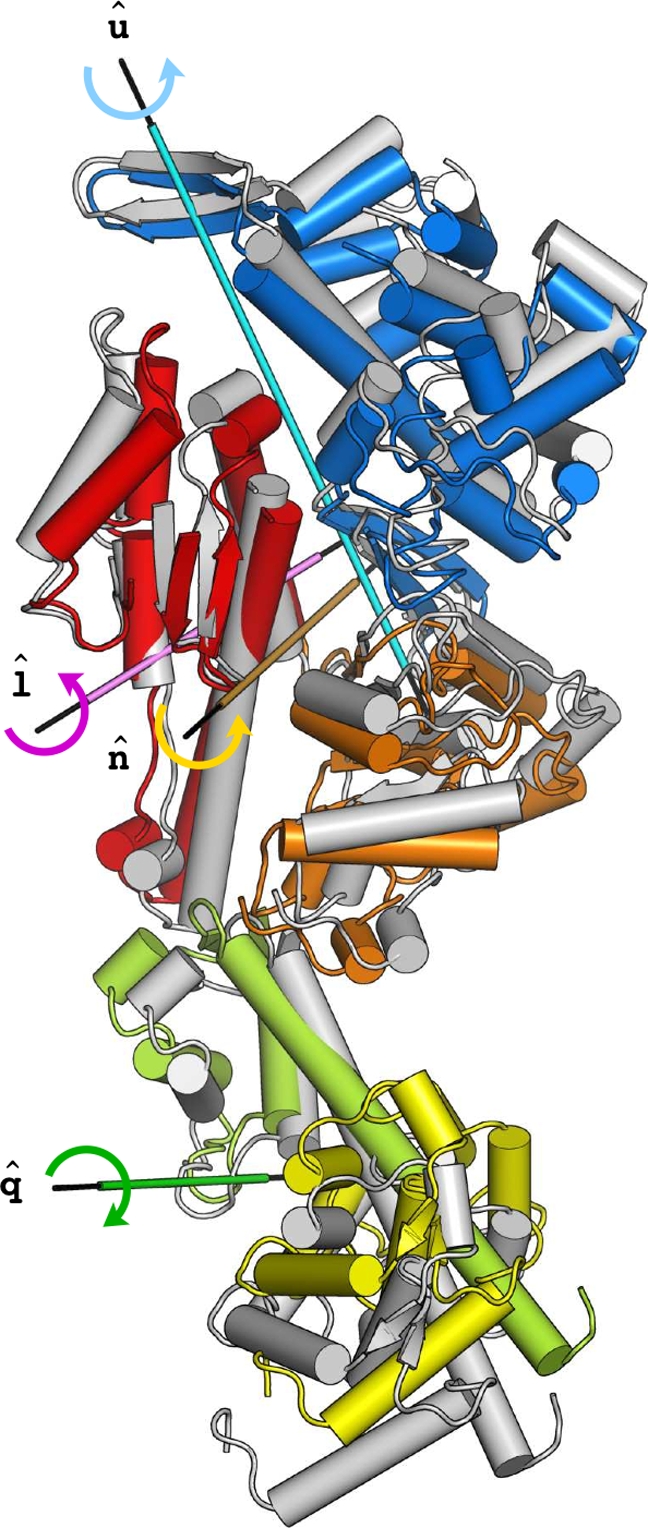
The rigor-like to NMSM post-rigor conformational transition. The energy-minimized rigor structure is color-coded as in Figure 2. The NMSM post-rigor conformation (referred to as 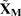 in the text) is shown in grey. The subdomain screw axes used to describe the transition in terms of individual subdomain rigid-body motions (see Table 3 and Text S4) are indicated; the screw axis corresponding to the C subdomain, ĉ, is omitted for clarity and shown in Figure 10E.

The overall rigid-body motion of the various subdomains is best described in terms of individual screw axes (see Figure 9, Table 3, and Text S4). As shown in Figure 9, **nˆ** and **lˆ** are oriented similarly and are almost orthogonal to **û**; i.e., **nˆ** and **lˆ** differ in orientation by only 38° and form angles of 83° and 89° with **û**, respectively. It follows that the N/L50 interface is preserved along the NMSM transition, while both the N/U50 and U50/L50 interfaces change substantially. As a result, the P-loop and switch I nucleotide-binding elements change their relative position and the U50/L50 cleft opens. The **lˆ** screw axis coincides with the one of the inertia axes of L50, suggesting that the contributions of U50 and L50 to the opening/closing of the cleft are different in nature; i.e., a rotation coupled to a translation describing a shear-like motion of the former and a pure rotation of the latter.

Visual examination of the NMSM transition pathway (see Video S1) shows a coordinated motion of the myosin motor subdomains in which the nucleotide-binding elements (P-loop and switch I) approach the binding site, the lever-arm is displaced downward, and the U50/L50 cleft opens. In the next subsections, the coupling between the large-amplitude motions of the individual subdomains, as described by the NMSM evolution of the rigor-like state, is analyzed in detail. We examine the pairwise (N/U50, N/C, L50/C, and N/L50) subdomain motions and their components. These rearrangements have important consequences concerning both the transmission and amplification of the allosteric signal.

### N/U50 Subdomains

**Figure 10:**
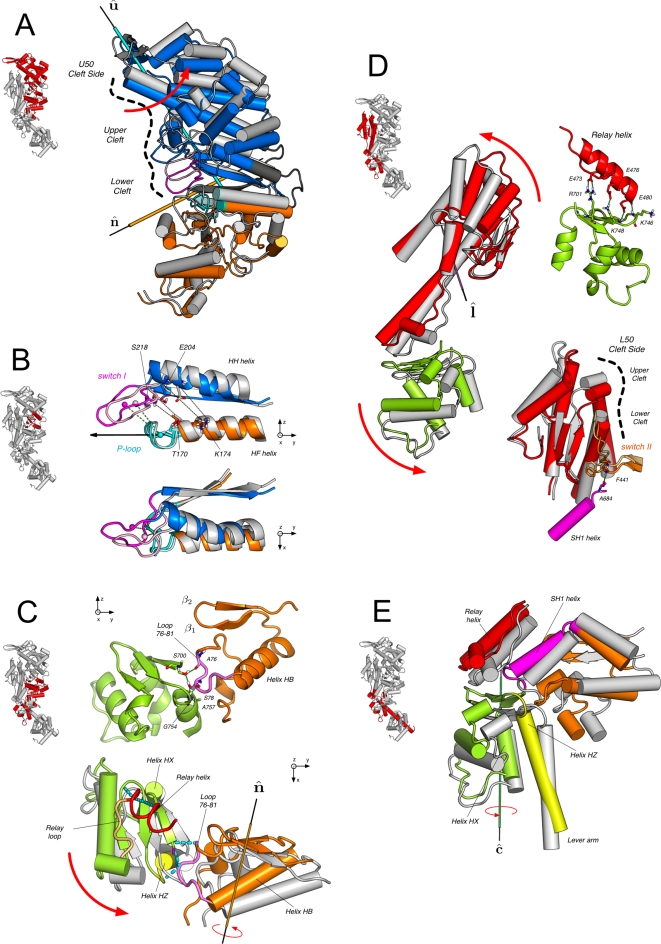
Structural rearrangement of the myosin subdomains along the rigor-like/post-rigor NMSM path; see also the corresponding sections in the text. The energy-minimized rigor-like structure is shown in colors; the NMSM post-rigor conformation is in grey with the nucleotide-binding elements shown in pale colors. The color code for the motor subdomains is the same as in Figure 2. The red arrows indicate the direction of motion of the subdomains along the NMSM transition. Insets on the left-hand side of each panel help to localize the structural elements which are being discussed. (A) N/U50 subdomains. Large-amplitude rotation of the N and U50 subdomains coupled to a local rearrangement in the ATP binding site; the screw axes û and nˆ are shown. (B) Structural transition of the nucleotide-binding elements. The two perpendicular views show the way switch I approaches the P-loop and moves “over” it. In doing so, the distance between Ser 218 and Thr 170, and Glu 204 and Lys 174 is substantially reduced (top), as reported in Table 4. In the ATP-bound state, the former pair of residues contributes to the coordination of the Mg^2+^ ion, while the latter pair makes a salt-bridging interaction. (C) N/C subdomains. On top is shown the network of H-bonds at the N/C interface responsible for the coupling. On bottom is shown the large-amplitude rotation of the N subdomain promoting the repositioning of the converter. The movement of the N-terminal is transmitted to the converter by specific interactions (shown as cyan dashed solid lines) involving Loop 76–81 (in violet). (D) L50/C subdomains. On the left are shown the large-amplitude motions of the converter and the L50 subdomain, which contribute to the opening of the U50/L50 cleft; on the right are shown the specific interactions involving the relay helix responsible for the L50/C coupling (on top) and the effect of the motion of L50 on the position of switch II (on bottom). The large-amplitude rotation of the L50 subdomain, which completes the opening of the U50/L50 cleft, is coupled to a rigid-body movement of switch II that breaks the rigor-like H-bonding interaction (Phe 441 - Ala 684) with the SH1 helix (see Table 4). (E) Reorientation of the lever arm. The lever arm, the relay helix, and the SH1 helix are shown in yellow, red, and magenta, respectively. Along with the orthogonal view (see panel C, on bottom), the picture shows how the displacement of the N subdomain is transmitted to the converter and transformed into a torque about axis ĉ that reorients the lever arm. The analysis suggests that the “short swinging” of the lever arm as observed in the X-ray structures [36] is a consequence of the rigor-like/post-rigor displacement of the converter.

The rotations of the N and U50 subdomains described by the NMSM evolution lead to a striking rearrangement of the relative position of the P-loop and switch I (see Figure 10, A and B); both the P-loop (part of N) and switch I (part of U50) tend to move as rigid bodies with the corresponding subdomains (see Text S5). This, perhaps, rather surprising result is one of the essential requirements for the allosteric transition. The rearrangement of the P-loop and switch I elements is best described by monitoring their change in position and orientation along the NMSM path (see Video S2). As listed in Table 4, the distance between their centers of mass is reduced by 0.7 Å. At the same time, the angle between **Pˆ**/**swI** (the vector linking the centers of mass) and **HĤ** **F** (the direction of the axis of helix HF) increases by 6°. In doing so, the P-loop and switch I move in the post-rigor direction to a position that would permit them to interact with the triphosphate moiety of ATP, presumably increasing the nucleotide-binding affinity. As shown in Figure 10B, switch I approaches the P-loop and moves “over” it. The further displacement of the P-loop (2.1 Å) that is required to complete the rigor-like/post-rigor transition is not found in the NMSM state. This local rearrangement is expected to be induced by the interaction with ATP in the post-rigor state.

**Table 4:** Observables that serve to monitor the allosteric mechanism which links ATP-binding to the opening of the U50/L50 cleft.

Subdomains pair	Elements involved	Observable	Description	Rigor	Rigor	Post	Post
				X-ray	min	NMSM	X-ray
**N/U50**	P-loop/Switch I	Distance (Å)	C.O.M.–C.O.M.	11.9	11.8	11.2	11.4
		Angle (deg)	**Pˆ**/**swI**·**Ĥ** **F**	39.4	42.3	48.7	67.9
	HF/HH	Distance (Å)	Lys 174/Cα–Glu 204/Cα	15.3	14.5	11.7	11.5
	Mg^2+^ site	Distance (Å)	Ser 218/Cα–Thr 170/Cα	11.2	10.3	8.9	7.8
	Adenosine pocket (aa 111–119, 99–100, 171, 175)	Cα-RMSD (Å)	from 1W7J	0.6	0.6	0.9	0.0
		Ca-RMSD (Å)	from 1W7I	0.5	0.5	1.0	0.2
	β-Sheet	Angle (deg)	β-Sheet (*τ*)	107.5	108.7	94.2	93.3
**N/C**	Loop 76–81/Converter	H-bond (Å)	Ala 76/O–Ser 700/Oγ	4.6	2.8	3.2	9.4
			Ser 78/Oγ–Ser 700/Oγ	4.4	2.8	2.9	9.2
			Ser 78/Oγ–Ala 757/N	3.0	2.9	3.1	7.4
			Ser 78/O–Gly 754/N	3.0	2.9	2.9	5.6
**L50/C**	Relay helix/Converter	H-bond (Å)	Glu 473/Oε_1_–Arg 701/NH_1_	2.9	2.8	3.0	5.2
			Glu 473/Oε_2_–Arg 701/NH_2_	2.7	2.8	2.8	3.0
			Glu 476/Oε–Lys 748/NZ	4.2	2.9	2.9	5.5
			Glu 480/Oε–Lys 748/N	2.9	3.0	2.9	3.0
			Glu 480/O–Lys 746/N	2.9	3.1	2.8	2.9
	Switch II/SH1		Phe 441/N–Ala 684/O	3.0	2.9	3.4	4.9

Helices HF and HH, which are structurally linked to the P-loop and switch I, respectively, allow the repositioning of the nucleotide-binding elements by a rigid-body movement that modifies their relative position and orientation. As a result, Thr 170 (on HF, part of N) and Ser 218 (on switch I, part of U50) move in the post-rigor direction (see Table 4) and the distance between them decreases; the distance between their C_α_ atoms is 11.2 Å in rigor-like and 7.8 Å in post-rigor. These two residues directly contribute to the coordination of the Mg^2+^ ion in the ATP-bound state of myosin [42]. Further, the relative displacement of helices HF and HH brings Lys 174 (on HF) and Glu 204 (on HH) closer (see Figure 10B). These two residues form a salt-bridge in the post-rigor state that is not present in the rigor-like state; the H-bond donor/acceptor distances are 12.1 and 2.6 Å in rigor-like and post-rigor, respectively. This interaction is expected to stabilize the post-rigor conformation of the myosin active site.

The effect of the transition on the binding site for the adenosin moiety, i.e., the portion of ATP composed of the adenine ring and the ribose sugar, was also investigated. Overall, the observed rearrangements of the amino acids contributing to the adenosine pocket in the X-ray structures are very small; i.e., a C_α_-RMSD of 0.6 Å is observed between the rigor-like and post-rigor states. This small change is not captured by the NMSM transition vector; the RMSD between the adenosine pocket in the X-ray and NMSM post-rigor structures is slightly larger than that between the X-rays rigor-like and post-rigor (see Table 4). The analysis suggests that the change of this portion of the ATP site (the adenosin pocket) is local, i.e., involves higher-frequency modes, and is not expected to have allosteric effects. This result supports the conclusion that the rigor/post-rigor conformational transition is triggered by the binding of Mg^2+^ and the triphosphate moiety of ATP.

A consequence of the large-amplitude reorientation of the U50 subdomain is a small upward movement of switch II. The shear-like motion of U50 results in the displacement of the fifth strand of the *β*-sheet (*β*_5_, part of U50), which connects to switch II and leads to its upward movement. Further, part of switch II extends *β*_5_ by two additional H-bonds with *β*_4_ (part of N) in the rigor-like state [16]; these are lost in the post-rigor state. Note also that the “rigid-body” movement of switch II results in the breaking of the interaction with the SH1 helix present in the rigor-like state (see below).

### The Central *β*-Sheet

**Figure 11:**
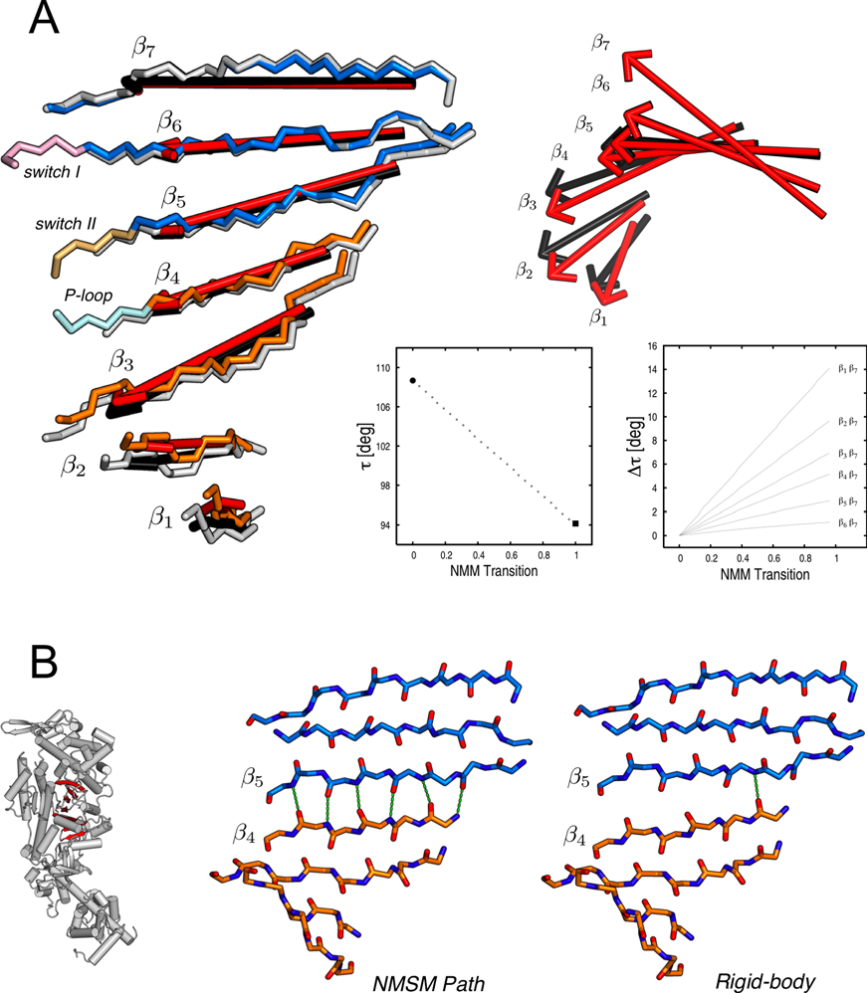
Structural rearrangement of the 7-stranded β-sheet. (A) Side and top views of the central β-sheet are shown on the left- and right-hand side of the panel, respectively. Individual β-strands, β*_i_*, are represented by red and black arrows indicating the direction of the rigor-like and NMSM post-rigor β-strand vectors, respectively. In the box diagrams, the twist of the entire sheet, *τ*, and the difference in the twist angle between the individual strands and β_7_, Δ*τ_i_*_,7_, relative to the rigor-like conformation are monitored along the NMSM path. The former is computed as 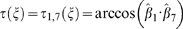, where 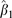 and 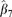 are the unit vectors corresponding to the structural borders of the β-sheet; the latter as Δ*τ_i_*_,7_(*ξ*) = ∥*τ_i_*_,7_(*ξ*)−*τ_i_*_,7_(0)∥, where *ξ* = 0 indicates the rigor-like conformation and *ξ* = 1 the NMSM post-rigor conformation. (B) The comparison of the NMSM post-rigor conformation with the structure obtained by the pure rigid-body motion described by the screw axes given in Table 3; see text. The inset on the left shows the structural location of the central *β*-sheet in the myosin head.

The NMSM transition path shows that the N/U50 large-amplitude rearrangement is associated with a partial untwisting of the 7-stranded *β*-sheet that structurally connects the two subdomains; strands *β*_1_–*β*_4_ are part of N, strands *β*_5_–*β*_7_ are part of U50 [14]. Given its location in the core of the motor domain and its large size, the myosin *β*-sheet connects several important elements and has been suggested to play an important role in the allosteric communication between the various functional sites [16]. Three *β*-strands are directly linked to the nucleotide-binding elements: *β*_4_ to the P-loop, *β*_5_ to switch II, and *β*_6_ to switch I (see Figure 11A). Thus, there is a coupling between the internal rearrangements of the *β*-sheet and the relative position and orientation of the P-loop, switch I and switch II. If the individual strands are represented as unit vectors, whose direction is determined by fitting their C_α_ atoms, the twist of the entire sheet, *τ*, can be computed as the inverse cosine of the dot product between 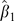 and 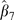, i.e., the vectors corresponding to the structural borders of the sheet. The twist angle, τ, is shown as a function of the fraction of the NMSM transition in Figure 11A (box diagrams). The total untwisting of the 7-stranded *β*-sheet corresponds to a Δ*τ* equal to 14.5°. Almost the same Δ*τ* value is obtained by comparing the two X-ray structures (i.e., *τ* is equal to 107.5° and 93.3° in rigor-like and post-rigor, respectively), indicating that the rigor-like/post-rigor transition of this key structural element is encoded in the low-frequency modes. The movement of the individual strands along the NMSM path can be described by their Δ*τ* relative to the starting conformation. As shown in Figure 11A, the relatively small Δ*τ* values observed between neighboring strands (e.g., Δ*τ* between 1.1° and 4.0°, see Table S7) are additive and result in the observed large flattening of the *β*-sheet.

To determine the origin of the partial untwisting of the *β*-sheet, which is energetically unfavorable [43],[36], the actual NMSM post-rigor conformation was compared with the structure obtained by pure rigid-body motions, as described by the screw axes given in Table 3 (see Text S4). Although the overall *β*-sheet twist (i.e., that between *β*_1_ and *β*_7_) is the same in both structures, Δ*τ* is restricted to strands *β*_4_ and *β*_5_ in the rigid-body transformation with a dramatic effect at the N/U50 interface; strands *β*_1_–*β*_4_ in N, and strands *β*_5_–*β*_7_ in U50 have Δ*τ_ij_* = 0, and Δ*τ*_45_ is equal to 13.7°. As shown by Figure 11B, the rigid-body motion of the N and U50 subdomains results in the loss of five out of six main-chain H-bonds between *β*_4_ and *β*_5_. This would correspond to an energetic penalty on the order of 15 to 20 kcal/mol [44] since the hydrogen bonds would not be replaced by water hydrogen bonds in the interior of the protein. By contrast, the energy cost of partial untwisting of the *β*-sheet is expected to be on the order of a few kcal/mol [45]. In the NMSM description of the untwisting, all the strands of the *β*-sheet are involved and no H-bonds are lost by standard criteria. Thus, the untwisting of the *β*-sheet appears to be an “adaptation” to avoid a large energy penalty from the relative motion of the U50 and N subdomains induced by the P-loop and switch I displacements.

### N/C Subdomains

The large-amplitude rotation of the N subdomain, which is a consequence of the repositioning of the P-loop, has important effects on the position/orientation of the converter (Figure 10C). The tight coupling between these two subdomains is such that the rotation of the N-terminal is transformed into a coupled translational and rotational movement of the converter, as evident from Table 3; i.e., both *ϕ* and *d* values for the converter are large in magnitude. The communication between the N and C subdomains in the rigor-like state is enabled by a short and rather rigid loop (aa 76–81; referred to as loop 76–81) that is involved in a complex network of H-bond interactions at the N/C interface (see Figure 10C). These H-bonds are listed in Table 4; in the post-rigor state these contacts are lost. The key interactions are formed between residues of the loop 76–81 and three residues on the converter; the former are Ala 76 and Ser 78, the latter Ser 700, Gly 754, and Ala 757. They are all part of secondary-structure elements that allow for efficient structural communication: Ser 700 is part of a quite rigid three-stranded *β*-sheet (see Table S8), and Gly 754 and Ala 757 belong to helix HZ, the last helix of the converter. The analysis thus assigns an important functional role to loop 76–81, as a connector of the N and C subdomains responsible for their coupling. The effect of such a coupling is two-fold: on the one hand, the translation of the converter promotes a large-amplitude rotation of the L50 subdomain; on the other hand, the rotation of the converter reorients the lever arm (see below).

### L50/C Subdomains

The translational movement of the converter is transmitted to the L50 subdomain by specific electrostatic interactions at the L50/C interface (see Figure 10D). The proximity of three negatively charged residues located on the relay helix (Glu 473, Glu 476, and Glu 480), and three positively charged residues located on the three-stranded *β*-sheet of the converter (Arg 701, Lys 746, and Lys 748) suggest that these amino acids are crucial for the L50/C coupling; as reported in Table 4, these residues contribute two salt bridges and two H-bonding interactions. Given these interactions, the L50 subdomain follows the downward movement of the converter which induces a large-amplitude rotation of L50 (Figure 10D). This motion is facilitated by the rigid-body movement of switch II initiated by the shear-like motion of U50 (see above) and completes the opening of the U50/L50 cleft. Switch II moves upwards by 0.5 Å and breaks the H-bonding interaction between Phe 441 (part of switch II) and Ala 684 (part of the SH1 helix) leading to the full opening of the cleft.

### N/L50 Subdomains

The interface of the N and L50 subdomains is essentially preserved along the NMSM path; the angle of 38° between the corresponding screw axes is too small to observe important effects at the subdomains interface (see **nˆ** and **lˆ** in Figure 9). Helix SH1, which couples the converter to switch II in the rigor-like state by a specific H-bond involving Phe 441 and Ala 684, lies at the N/L50 interface; it is not part of either subdomain. The rigor-like position of helix SH1 is further stabilized by multiple hydrophobic contacts with the side chains of neighboring amino acids [16], i.e., residues 75, 80, 82, and 85 on the N-terminal, and residues 488 and 490 part of the relay loop on L50. None of these contacts is lost along the NMSM transition path. The breaking of the H-bond between Phe 441 and Ala 684 (see above) is an important factor in permitting the large-amplitude rotation of L50 that is coupled to a rigid-body movement of the group formed by the relay loop, the SH1 helix, and the N subdomain. The reorientation of the SH1 helix along with a local deformation of the region connecting the helix SH1 to the converter (aa 695–699), which acts as a hinge, results in the observed downward movement of the latter. This movement weakens the coupling between the converter and the head domain.

The NMSM path suggests that the breaking of the H-bond with switch II is the key element in the release of the converter. Since the “sliding” of the SH1 helix between the relay loop and the N-terminal does not occur in the NMSM state, it appears not to be directly involved in the release of the converter. Rather, the change in the interaction pattern of the SH1 helix with its surroundings, as observed in comparing the rigor-like and post-rigor structures [16], is a more local event likely to contribute primarily to stabilizing the post-rigor conformation.

### Reorientation of the Lever-Arm

The rigor-like to post-rigor transition promotes a small reorientation of the lever arm; a swinging motion of ∼14°, as observed from the X-ray structures. The NMSM transition pathway suggests that this small “swing” is a consequence of the allosteric communication between the N, C, and L50 subdomains. The large-amplitude rotation of the N-terminal is coupled to a translational movement of the converter, as described above. The tight coupling between the converter and L50 constrains the conformational freedom of the former and transforms the translational input from N into a torque about helix HX (see Figure 10E). This results in a large-amplitude rotation of the converter that reorients the lever arm, which is the structural prolongation of the converter helix HZ.

## Discussion

The purpose of this paper is to obtain an understanding of the rigor to post-rigor transition in myosin V. This transition is an essential part of the functional cycle because it is directly involved in going from a strongly bound actomyosin complex (after the powerstroke) to the separated actin and myosin molecules, in preparation for the re-priming of the latter. The “trigger” of the dissociation is the binding of ATP to myosin and the resulting opening of the actin-binding cleft. A fundamental question concerns the allosteric mechanism by which the local changes induced by ATP binding to myosin lead to the global change involving opening of the cleft and lever-arm uncoupling. The present paper uses a block normal mode analysis of the rigor-like and post-rigor functional states of myosin V to obtain insights into this mechanism.

The intrinsic flexibility of the myosin molecule, as described by the low-frequency normal modes, was investigated in the two functional states. Despite the large-amplitude conformational change (i.e., the C_α_-RMSD between the two structures is 5.2 Å) the low-frequency modes are very similar in the rigor-like and post-rigor states, pointing to their “robustness” [40]. The modes identify two dynamic domains: the “head” or motor domain that is responsible for ATP hydrolysis and actin binding, and the “neck” region connecting the lever arm to the myosin head. In both functional states, the three lowest-frequency modes correspond to the motion of the neck relative to the head, with essentially no internal rearrangements in either domain. The dynamic behavior of the converter subdomain, which is located at the interface between the head and neck domains, differs between the rigor and post-rigor states; i.e., the dynamic domains obtained from the modes show that the converter belongs to the head in the rigor-like state and to the neck in post-rigor; in fact, the converter is only weakly coupled to the head domain in post-rigor. Thus, the post-rigor converter appears free to move to the pre-powerstroke state to “re-prime” the lever arm, the next conformational transition in the actomyosin cycle. This conclusion is in accord with experimental and simulation results indicating that, in the post-rigor state with ATP bound, there is an equilibrium between the post-rigor and pre-powerstroke positions of the converter [18],[19]; the latter is stabilized by the presence of the hydrolysis products, ADP and P_i_.

Analysis of the lowest-frequency modes, which dominate the lever-arm displacements, showed that the lever-arm orientation is essentially uncoupled from the head domain in both states. In accord with the above paragraph, the hinge region defining the structural boundaries of the head and neck domains is located differently in the two structures; i.e., the hinge region is at the junction between the converter and the lever arm in the rigor-like state, and at the end of the SH1 helix (i.e., before the converter) in post-rigor, as shown in Figure 6B. Thus, the lever arm appears to be uncoupled from the converter in the rigor-like state and to move with it in the post-rigor state. The uncoupling in the rigor-like state makes possible the reorientation of the lever arm without internal reorganization of the motor domain. Moreover, the lever arm can move easily in perpendicular directions. This freedom of movement, as well as the observed twisting motion, is likely to be involved in the hand-over-hand stepping of myosin V on actin [2],[3]. Possible examples occur when the rigor state is present with both heads bound to actin and dissociation of ADP from the trailing head has preceded the strong binding to actin of the leading head [12],[46]. The flexibility of the lever arm in the trailing head may be required to absorb stress introduced by the powerstroke of the leading head and prevent dissociation from actin. Also, in the forward step, flexibility may be required in the trailing head to permit the motor domain of the leading head to rebind to actin. Despite the enhanced flexibility observed in the post-rigor state, the converter is not free to “spin”. To acquire such conformational freedom, something like the “detached state” observed for the scallop myosin head would be required [47]. Unwinding of the SH1 helix in this state leads to a converter that is essentially free to rotate.

During the powerstroke when myosin is bound to actin, a force is transmitted from the motor domain to the lever arm. The lever arm must therefore be coupled to the motor domain in this step of the cycle. Given the analysis described above, the required coupling appears not to be present in the rigor-like state, which represents an actin-bound state. Whether the coupling is already present in the pre-powerstroke state or occurs later in the cycle, possibly as a consequence of structural changes induced by the release of the hydrolysis products and/or by actin binding is not known; the intrinsic flexibility of the myosin molecule in the pre-powerstroke state of various myosins is being studied (MC and MK, in progress).

The next set of low-frequency modes provides a description of the rearrangements within the head domain in going from the rigor-like to the post-rigor state. Approximately ten additional modes are required for the global motions of the subdomains, although a larger number (around 40) are needed for a detailed representation of the transition pathway. The normal mode analysis suggests that the primary effect of ATP is to stabilize the post-rigor structure, rather than to alter the intrinsic myosin flexibility. There is a small change in flexibility as a consequence of nucleotide binding as indicated by the slightly different activation of the modes in the two myosin states.

The involvement coefficients projected on specific structural elements demonstrate that several modes contribute to the structural rearrangements in all cases; i.e., no individual mode gives an adequate description of the complete transition pathway for any structural element. For example, the rearrangement at the U50/L50 interface, which is an essential part of the opening/closing of the cleft, involves at least three modes. The same is true for the N/U50 and L50/C interfaces, whose rearrangements are associated with ATP binding and the structural communication between the actin-binding site and the lever arm; they require three and four modes, respectively. This confirms that analyses of conformational transitions of myosin [32],[33] and other proteins [29],[48] based on a single mode with a large involvement coefficient provide at best a rather crude description [49]–[51].

**Figure 12:**
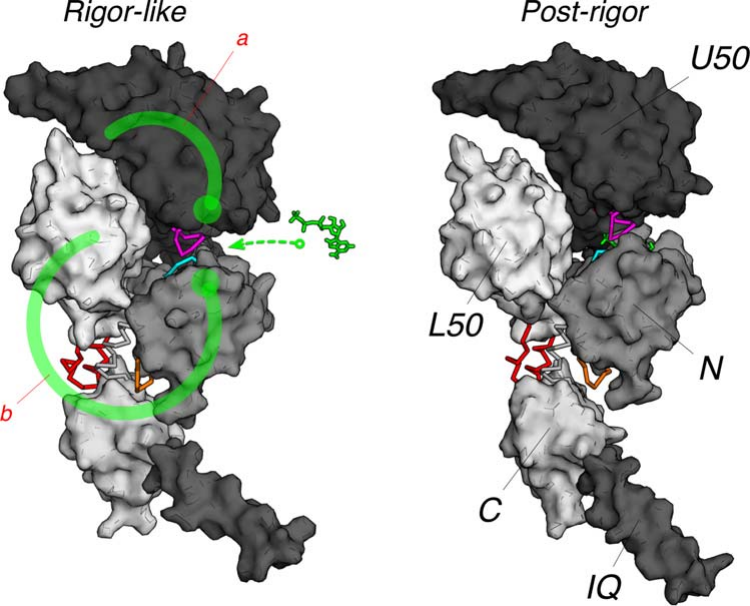
Molecular mechanism of the rigor-like to post-rigor transition as described by the NMSM pathway. The molecular surface of the myosin subdomains is shown in tones of grey; the nucleotide is in green. Key residues at the subdomain interfaces that are responsible for the coupling between the myosin subdomains are depicted in cyan, magenta, orange, and red; they correspond to the P-loop, switch I, loop 76–81, and the relay helix, respectively. These allosteric connectors couple the local changes due to ATP binding to the more global motions of the myosin molecule. The ATP-binding signal is transmitted to the U50/L50 cleft through two distinct communication pathways (shown as heavy green lines; the large dot indicates the approximate origin of the signal). Path a involves the U50 subdomain and is consistent with the interpretation of three-dimensional electron cryo-microscopy reconstructions by Holmes et al. [60]. Path b involves the N-terminal subdomain, the converter and the L50 subdomain. In the latter, the transmission of the ATP binding signal is the consequence of the coordinated movement of the three subdomains described by the NMSM path (see Video S1). The allosteric communication results in the opening of the U50/L50 cleft and the uncoupling of the converter from the motor head.

Given the above, we describe the rigor-like/post-rigor transition path by an optimal superposition of the required number of low-frequency modes, as determined from the involvement coefficients. By using 40 modes of the rigor-like state (out of the total of 5,448 in the block normal mode approximation) the optimal superposition results in a conformation that is close to the post-rigor structure. It has a C_α_-RMSD of only 1.28 Å as compared with the rigor-like/post-rigor C_α_-RMSD of 5.2 Å. We refer to this structure as the normal mode superposition model (NMSM) conformation. The NMSM path (i.e., the one from the rigor-like to the NMSM conformation) shows a highly coordinated set of movements that provides new insights concerning the mechanism of the rigor/post-rigor conformational change. It demonstrates that the coupling between the *local* structural changes due to ATP binding and the *global* motions of the protein encoded in these low-frequency modes leads to the allosteric transmission required for the amplification of the signal. In particular, the path shows the nature of the coupling between the myosin subdomains and offers a rational explanation for the mutual exclusion of ATP and actin binding. The rearrangement of the P-loop and switch I is the triggering event, in which the two nucleotide-binding elements come close to each other and move in the post-rigor direction so as to be able to interact with ATP. This local change initiates the more global motions in the myosin head. The NMSM path shows two distinct structural communication pathways coupling the nucleotide-binding site to the U50/L50 cleft. The immediate structural consequence of the switch I motion is a partial opening of the cleft on the U50 side, while the P-loop motion leads to a large-amplitude rotation of the N subdomain. The tight coupling of the N-terminal subdomain to the converter through loop 76–81, which belongs to the former and forms H-bonds to residues in the latter, results in a translational/rotational movement of the converter as a consequence of the rotation of the N subdomain. The translation of the converter promotes a large-amplitude rotation of the L50 subdomain that leads to the complete opening of the U50/L50 cleft, presumably followed by the detachment of myosin from actin. The rotation of the converter coupled to its translational movement causes the lever arm to move downward by a small rotation (swinging) in a direction almost orthogonal to the recovery-stroke, as observed in various myosin X-ray structures [36]. The position of the converter appears to be controlled by a main-chain hydrogen bond between switch II (Phe 441) and the SH1 helix (Ala 684). During the transition to post-rigor this H-bond is lost as a consequence of a small upward movement of switch II; i.e., the rigid-body movement of switch II is initiated by the displacement of *β*_5_ coupled to the shear-like motion of U50, and is completed by the large-amplitude rotation of L50 that opens the cleft. The uncoupling of the SH1 helix from switch II results in a large downward movement of the converter which is released from the motor domain and becomes ready for the transition to the pre-powerstroke state. This completes the rigor to post-rigor transition (Figure 12).

The molecular mechanism described by the NMSM model proposes an important role for the converter subdomain in the allosteric communication between the nucleotide and actin binding sites. Mutations that either weaken the coupling between the converter and both the N-terminal and the L50 subdomain, or block the converter in its rigor-like position are expected to hinder the rigor/post-rigor transition and thereby alter the release from actin. In particular, two residues at the N/C interface (i.e., Ser 78 and Ser 700) and six residues at the L50/C interface (i.e., Glu 473, Glu 476, Glu 480, Arg 701, Lys 746, and Lys 478), which are all involved in strong interactions with the converter appear to be good candidates for mutagenesis. Cross-linking switch II to the SH1 helix by engineering specific disulfide bridges is an alternative suggestion. For this type of approach, Phe 441 (part of switch II) and Ala 684 (part of SH1 helix) appear to be the best candidates.

The key H-bonding interaction between switch II and helix SH1 is not present in the available rigor-like structures of myosin II [36],[52] or myosin VI [53]. In rigor-like myosin V, this H-bond is allowed by the “down” position of switch II that corresponds to the largest extent of cleft closure that has been observed [36]. By stabilizing the unique position of switch II, this interaction facilitates the large-amplitude rotation of the L50 subdomain, allows a fully closed cleft, and “locks” the converter in its rigor-like position. Thus, this hydrogen bond is expected to increase the conformational stability of the strong actin-binding states of myosin and play a role in the processive nature of myosin V.

An important structural change that has been noted by others in the rigor/post-rigor conformational transition [15],[16] is the flattening (partial untwisting) of the 7-stranded β-sheet, which is part of N and U50 subdomains; the crystal structures show an untwisting by about 14° (from 107.5° to 93.3°). The NMSM evolution suggests that the observed untwisting is highly cooperative and all strands significantly contribute to the difference in twist. The contributions of pairs of neighboring strands are small but additive, leading to the overall flattening. In going from the upper (*β*_7_, close to the active site and part of U50) to the lower (*β*_1_, far from the active site and part of N) boundaries of the *β*-sheet along its longitudinal axis, the untwisting corresponds to increasing displacements of the individual strands. Such an untwisting, or flattening, is caused by the fact that, if the *β*-sheet rigidly followed the N and U50 rotation, at least five H-bonding interactions at the N/U50 interface would be lost. To retain the hydrogen bonds the *β*-sheet partly untwists and stores the energy associated with its untwisting. This analysis is in agreement with the qualitative description of Yang et al. [36] based on visual comparison of different myosin II structures. Once binding to actin has taken place, partial switch II opening results in phosphate release, which is thought to be required for the powerstroke, at least in myosin V. If the powerstroke involves both the P-loop and switch I moving back to their rigor-like position under the action of actin, the energy stored in the *β*-sheet could be released as the latter assumes its low-energy fully twisted conformation. At this stage of the cycle, a force can be actively transmitted to the converter, so that the *β*-sheet now works as a “transducer”, as previously suggested [16]. According to this interpretation, the *β*-sheet acts primarily as an “adapter” in the free myosin transitions corresponding to the recovery-stroke (rigor/post-rigor and post-rigor/pre-powerstroke) and as a “transducer” in the actin-binding states associated with the powerstroke.

The description of the transition is in accord with the allosteric concept that the initial (rigor-like) and the NMSM post-rigor-like structure are in equilibrium [54], with the former lower in free-energy than the latter in the absence of ATP. Once ATP binds, the free-energy surface is altered so that the NMSM structure, as well as the actual post-rigor structure, becomes lower in free energy. It is possible, in analogy with the suggestion for other allosteric systems [55]–[57], that ATP binding occurs to a higher free-energy NMSM-like conformation, which has only a small population in the absence of ATP, but in its presence is transformed to the now more stable post-rigor state. In this scenario, the role of ATP would be to capture the fluctuations of the nucleotide-free myosin that promote the rigor to post-rigor conformational transition. It is possible, also, that partial ATP binding occurs already to the rigor-like conformation accompanied by local changes in the P-loop. ATP would then be able to recruit switch I and force its closing movement via long-range electrostatic interactions. In this alternative scenario, the role of the interactions between myosin and the nucleotide would be to optimally select and combine the low-frequency modes naturally encoded in the protein to promote the conformational transition. Which of these two possibilities actually occurs could be different for free myosin and myosin bound to actin since the fluctuations (i.e., those leading to the NMSM state) are expected to be more restricted in the latter than in the former; also, the equilibrium between the rigor and post-rigor states would be shifted toward the former in the bound state. Further experiments and simulations are required to resolve this question.

## Supporting Information

Video S1 The NMSM transition pathway. The movie shows the rigor to post-rigor transition as described by the optimal superposition of the 40 lowest-frequency rigor modes. The rigor (thick tube) and the post-rigor (thin tube) structures are shown in colors and grey, respectively. The color code is as follows: the N, U50, L50, and C/IQ subdomains are colored in orange, blue, red, and lime, respectively; the P-loop, switch I, switch II, SH1 helix, and loop 76–81 connectors are colored in cyan, magenta, yellow, wheat, and pink. The movie shows a coordinated motion of the myosin motor subdomains in which the nucleotide-binding elements (P-loop and switch I) approach the binding site, the lever-arm is displaced downward, and the U50/L50 cleft opens.
(3.62 MB MOV)

Video S2 The NMSM rearrangement of the P-loop and switch I. The movie shows the rigor to post-rigor rearrangement of the P-loop and switch I nucleotide-binding elements as described by the optimal superposition of the 40 lowest-frequency rigor modes. The rigor (thick tube) and the post-rigor (thin tube) structures with the N-terminal subdomain aligned are shown in colors and grey, respectively. The ATP molecule in the post-rigor structure is shown in white as a stick representation. The color code is as follows: the N and U50 subdomains are colored in orange and blue; the P-loop, switch I, and switch II linkers are colored in cyan, magenta and yellow, respectively. The movie shows the way switch I approaches the P-loop and moves “over” it to coordinate the nucleotide.
(10.17 MB MOV)

Text S1 Preparation of the structures.
(0.10 MB PDF)

Text S2 Normal mode calculation and analysis.
(0.10 MB PDF)

Text S3 Normal mode analysis of the rigor to apo post-rigor transition.
(0.16 MB PDF)

Text S4 Screw axis determination.
(0.72 MB PDF)

Text S5 Flexibility of the subdomain linkers.
(0.07 MB PDF)

Table S1 Myosin V subdomains and linkers.
(0.03 MB PDF)

Table S2 Myosin V secondary-structure elements.
(0.04 MB PDF)

Table S3 Rigor-like and post-rigor normal mode overlaps.
(0.03 MB PDF)

Table S4 Rigor-like and post-rigor normal mode frequencies.
(0.03 MB PDF)

Table S5 Involvement coefficients specialized for the rigor-like/post-rigor transition of the motor domain and individual subdomains.
(0.03 MB PDF)

Table S6 Inter-subdomain center of mass distances from the rigor-like to the post-rigor and NMSM structures.
(0.03 MB PDF)

Table S7 Rigor-like/NMSM Δτ values between pairs of neighboring strands of the central β-sheet.
(0.03 MB PDF)

Table S8 Flexibility of the converter β-sheet.
(0.04 MB PDF)

Figure S1 Center-of-mass displacement of the motor subdomains along the rigor to NMSM post-rigor transition. The rigor structure is shown in colors, the NMSM post-rigor conformation in grey. Black and red spheres indicate the position of the subdomains centers of mass in the rigor-like and the NMSM post-rigor conformation, respectively. The downward translation of converter (C) in the opposite direction to the translational motion of both N and U50 weakens the coupling between the neck region and the head domain.
(2.44 MB TIF)
