# Supplementary material for: Allosteric Communication in Myosin V: From Small Conformational Changes to Large Directed Movements
Source: PLoS Comput Biol. 2008 Aug 15;4(8):e1000129. doi: 10.1371/journal.pcbi.1000129 (PMC2497441; doi:10.1371/journal.pcbi.1000129)
Supplement: Text S1 — Preparation of the structures. (0.10 MB PDF) [file pcbi.1000129.s003.pdf]

Preparation of the Structures (Text S1)

Model

The proteins were modeled by explicitly considering all heavy atoms and the hydrogen atoms bound to nitrogen or oxygen atoms. The remaining hydrogen atoms were treated as part of the carbon atoms to which they are covalently bound (extended-atom approximation). The potential of the model used for all energy minimizations and normal mode calculations is the polar-hydrogen potential function (PARAM19) [1], as implemented in CHARMM [2]. In this representation, the effective energy function for a molecular system with N atomic nuclei located at $\mathbf{r} = (\mathbf{r}_1, ..., \mathbf{r}_N)$ is defined as

$$E(\mathbf{r}) = E_{vacuo}(\mathbf{r}) + \Delta G_{solv}(\mathbf{r}) \quad (1)$$

The *in vacuo* energy term is

$$\begin{aligned} E_{vacuo}(\mathbf{r}) \; = \; & \frac{1}{2} \sum_{bonds} k_b (b - b_0)^2 \; + \frac{1}{2} \sum_{\substack{bond \\ angles}} k_\theta (\theta - \theta_0)^2 \\ & + \; \frac{1}{2} \sum_{\substack{dihedral \\ angles}} k_\phi [1 + \cos(n\phi - \delta)] \\ & + \; \frac{1}{2} \sum_{\substack{improper \\ dihedrals}} k_\omega (\omega - \omega_0)^2 \\ & + \; \sum_{i>j} \varepsilon_{ij}^{\min} \left[\left(\frac{d_{ij}^{\min}}{r_{ij}} \right)^{12} - 2 \left(\frac{d_{ij}^{\min}}{r_{ij}} \right)^{6} \right] \\ & + \; \sum_{i>j} \frac{q_i q_j}{\epsilon(r_{ij}) r_{ij}} \end{aligned}$$

where b is a bond length, θ a bond angle, ϕ a dihedral angle, ω an improper dihedral, r_{ij} is the distance between atoms i and j, q_i and q_j are partial charges, and $d_{ij}^{\min}$ and $\varepsilon_{ij}^{\min}$ are the optimal van der Waals distance and energy, respectively; parameters are given in Ref. [1].

Solvation effects were approximated by the implicit solvent model EEF1 [3], which contains screened electrostatic interactions and a Gaussian term to represent full hydrophobic interactions. In this approximation, the solvation free energy can be decomposed into a sum of pairwise interactions

$$\Delta G_{solv}(\mathbf{r}) = \sum_{i=1} \Delta G_i^{\text{ref}} - \sum_{i=1} \sum_{j>i} f_i(r_{ij}) \, V_j \quad (2)$$

where ΔG_i^{ref} is the solvation free energy of the i-th atom in a suitably chosen small molecule so that the atom is essentially fully solvent-exposed, f_i is the solvation free-energy density around atom i, and V_j is the volume of the j-th atom around i. It follows from Eq. 2 that the solvation free energy of atom i is equal to its solvation free energy in a model system (ΔG_i^{ref}) minus the reduction in solvation due to presence of the surrounding groups. Thus, the model evaluates the solvation free energy of a given atom by computing the effect of its neighboring atoms, which exclude solvent from the surrounding space, on the solvation free energy density. By assuming that the solvation free-energy density around any atom is Gaussian distributed, the total solvation free-energy can be re-written as

$$\Delta G_{solv}(\mathbf{r}) = \sum_{i=1} \Delta G_i^{\text{ref}} - \sum_{j>i} \left\{ \frac{2\Delta G_i^{\text{free}}}{4\pi\sqrt{\pi}\lambda_i r_{ij}^2} \exp(-x_i^2) V_j + \frac{2\Delta G_j^{\text{free}}}{4\pi\sqrt{\pi}\lambda_j r_{ij}^2} \exp(-x_j^2) V_i \right\} \quad (3)$$

with

$$x_i = \frac{r_{ij} - R_i}{\lambda_i} \quad (4)$$

where for the i-th atom ΔG_i^{free} is the solvation free energy of the isolated atom, λ_i its correlation length, and R_i its van der Waals radius. Given Eqs. 3 and 4 the evaluation of the solvation free energy only requires the choice of atom type i, their volumes V_i, their correlation length λ_i, ΔG_i^{ref}, and ΔG_i^{free}. The specific atom types and solvation parameters were chosen as described in Ref. [3]. Ionic side chains were neutralized and a linear distance-dependent screening function ($\epsilon(r_{ij}) = r_{ij}$) was used for the electrostatic interactions. Both van der Waals and electrostatic interactions were cut off at 9 Å with a switching function used between 7 and 9 Å. The EEF1 solvation model has been successfully applied to a wide range of problems, including protein folding [4, 5], mechanical unfolding of proteins [6, 7], protein function investigation [8], molecular modeling and protein structure prediction [9], computational design and protein engineering [10, 11], and protein-protein docking [12].

Protein Structures

The initial coordinates of the myosin V molecule in the rigor-like and post-rigor functional states were downloaded from the Protein Data Bank [13] (PDB entries 1OE9 and 1W7J, solved at 2.05 and 2.00 Å resolution, respectively). The rigor-like structure is a nucleotide-free myosin conformation, while the post-rigor structure represents an ATP-bound state, as described in “Main Text”. Since the post-rigor conformation was solved in complex

with an ATP analog ($MgADP.BeF_3$), coordinates for ATP were not present in the X-ray structure. During the preparation, the ATP moiety was modeled by using the $ADP.BeF_3$ coordinates and existing force-field parameters [1]; i.e., the position of BeF_3 and the oxygen atom linking the berillium atom to P_β were used to place the missing phosphate group before the overall minimization. In both crystal structures a number of residues and segments were missing, i.e., 77 (64) residues were completely missing and 38 (77) residues were partially missing in the rigor-like (post-rigor) conformation and had to be introduced by a rather time-consuming procedure to obtain reliable structures. For the rigor-like structure, five missing segments (i.e., aa 1-4, 53-56, 185-190, 382-385, and 484-487) were introduced from a second X-ray rigor-like structure at slightly lower resolution and lacking all IQ motifs [14] (PDB entry: 1W8J, solved at 2.7 Å resolution). The remaining missing residues were modeled and the structure relaxed in the following way: hydrogens, missing residues with dihedral constraints on the ω angles, and the side chains were minimized with 5000 steepest descent (SD) and 10000 adopted basis Newton-Raphson (ABNR) steps with the positions of non-missing backbone atoms fixed. Then, for the completely missing residues a simulated annealing conformational search was performed by molecular dynamics while keeping the rest of the protein fixed; i.e., missing residues and the surrounding side chains were heated up within 50 ps to 800 K and kept at this temperature for 500 ps and then cooled down to 100 K in 1.4 ns. After the complete structure had been obtained, the protein backbone was relaxed by performing 20 minimization cycles, 1000 SD steps each, with harmonic constraints on the backbone. To guarantee a gentle relaxation of the structure, decreasing force constant values ranging from 10000 to 0.01 kcal/mol $Å^2$ were applied to the backbone atoms. The resulting structures were finally minimized with 1000 SD steps without constraints. At this stage, loop 2 (i.e., the largest missing loop in the original crystallographic structures that connects U50 and L50) was deleted. In myosin V, loop 2 is very long (38 aa) and flexible. It is supposed to mediate the interactions between actin and myosin, thus modulating the actin-binding affinity of myosin [15]. However, in the absence of actin it appears to be unstructured, as shown by several myosin V crystal structures where it is not resolved [14, 16]. Given its size and flexibility, keeping a modeled conformation of loop 2 in the normal mode calculations would be likely to introduce spurious results. Both rigor-like and post-rigor structures with loop 2 deleted were then minimized with 10000 SD and 10000 ABNR steps. During each minimization step a tolerance of 0.001 kcal/mol $Å^2$ was applied to the

average gradient. Such a gradient is sufficiently small not to have any negative eigenvalues (see below). The resulting energy-minimized conformations were used for the normal mode calculations. Both structures show small root-mean-square deviations (RMSD) from the original X-ray conformations (see Table I), so that they provide satisfactory representations of the myosin rigor-like and post-rigor states. The RMS difference between the rigor-like and post-rigor structures is 5.4 (5.2) Å for X-ray and 5.3 (5.2) Å after energy minimization; the all-atom result is given first and the C_α result in parentheses.

	all-atom RMSD	C_α-RMSD	Energy
**Rigor**	1.49	0.98	-28911.4
**Post-Rigor**	1.54	0.86	-29460.7

TABLE I: Analysis of the rigor-like and post-rigor conformations used for the BNM analysis. All-atom and C_α-RMSD values are given in Å. Effective energies, which include potential and solvation energy terms, are given in kcal/mol.

[1] Neria E, Fischer S, Karplus M (1996) Simulation of activation free energies in molecular systems. J Chem Phys 105:1902–1921.

[2] Brooks B, Bruccoleri R, Olafson B, States D, Swaminathan S, et al. (1983) CHARMM: A program for macromolecular energy, minimization, and dynamics calculations. J Comput Chem 4:187–217.

[3] Lazaridis T, Karplus M (1999) Effective energy function for proteins in solution. Proteins: Structure, Function and Genetics 35:133–152.

[4] Paci E, Vendruscolo M, Dobson C, Karplus M (2002) Determination of a Transition State at Atomic Resolution from Protein Engineering Data. J Mol Biol 324:151–163.

[5] Krivov S, Karplus M (2004) Hidden complexity of free energy surfaces for peptide (protein) folding. Proc Natl Acad Sci USA 101:14766.

[6] Paci E, Karplus M (2000) Unfolding proteins by external forces and temperature: the importance of topology and energetics. Proc Natl Acad Sci USA 97:6521–6526.

[7] Fowler S, Best R, Toca Herrera J, Rutherford T, Steward A, et al. (2002) Mechanical unfolding of a titin Ig domain: structure of unfolding intermediate revealed by combining AFM, Molecular Dynamics simulations, NMR and protein engineering. J Mol Biol 322:841–849.

[8] van der Vaart A, Ma J, Karplus M (2004) The unfolding action of GroEL on a protein substrate. Biophys J 87:562–573.

[9] Toward high-resolution de novo structure prediction for small proteins .

[10] Dantas G, Kuhlman B, Callender D, Wong M, Baker D (2003) A Large Scale Test of Computational Protein Design: Folding and Stability of Nine Completely Redesigned Globular Proteins. J Mol Biol 332:449–460.

[11] Korkegian A, Black M, Baker D, Stoddard B (2005) Computational thermostabilization of an enzyme. Science 308:857–860.

[12] Gray J, Moughon S, Wang C, Schueler-Furman O, Kuhlman B, et al. (2003) Protein–protein docking with simultaneous optimization of rigid-body displacement and side-chain conformations. J Mol Biol 331:281–299.

[13] Berman HM, Westbrook J, Feng Z, Gilliland G, Bhat TN, et al. (2000) The protein data bank. Nucl Acids Res 28:235–242.

[14] Coureux PD, Sweeney HL, Houdusse A (2004) Three myosin V structures delineate essential features of chemo-mechanical transduction. EMBO J 23:4527–4537.

[15] Yengo C, Sweeney HL (2004) Functional role of loop 2 in myosin V. Biochemistry 43:2605–2612.

[16] Coureux PD, Wells AL, Menetrey J, Yengo C, Morris CA, et al. (2003) A structural state of the myosin V motor without bound nucleotide. Nature 425:419–423.
