# Supplementary material for: Allosteric Communication in Myosin V: From Small Conformational Changes to Large Directed Movements
Source: PLoS Comput Biol. 2008 Aug 15;4(8):e1000129. doi: 10.1371/journal.pcbi.1000129 (PMC2497441; doi:10.1371/journal.pcbi.1000129)
Supplement: Text S2 — Normal mode calculation and analysis. (0.10 MB PDF) [file pcbi.1000129.s004.pdf]

Normal Mode Calculation and Analysis (Text S2)

Normal Mode Calculation

Normal mode analysis was performed on both functional states of myosin V by using the block normal mode (BNM) method [1, 2] with the EEF1 solvent parameterization [3]; this implicit solvent force field provides analytical second derivatives for the normal mode calculations. In the BNM approach, the molecule is partitioned into moving units (blocks, here each unit contains one residue) and the atomic mass-weighted Hessian, $\widetilde{\mathcal{H}}$, is projected onto the subspace spanned by the translation and rotation vectors of the blocks. Even though the internal degrees of freedom of the moving units are frozen in the BNM description, which limits the side chain motions, previous studies have shown that the results are appropriate for the investigation of the modes of primary interest for conformational changes [1, 2, 4]. The BNM calculations have the advantage that the computational cost is dramatically reduced without any additional approximation in the treatment of the atomic interactions, in contrast to elastic models [5]. BNM is also likely to give more robust results than NM calculations with all degrees of freedom for relatively low resolution (incomplete) structures. In the rigor-like and post-rigor BNM calculations 909 and 910 blocks were defined, respectively. They correspond to 5454 (26997) and 5460 (27096) degrees of freedom in the BNM approximation, respectively; the total atomic degrees of freedom are given in parentheses. The first 908 blocks include amino-acid residues of the heavy and light chains of myosin; the remaining blocks include a SO_4^{-2} ion in rigor-like, and ATP and Mg^{+2} in post-rigor. In both myosin states, the diagonalization of the blocked Hessian matrix to determine the BNM eigenvectors and eigenvalues took less than 30 minutes on a single 2.8 GHz Athlon. No negative eigenvalues were present in the BNM analyses, indicating that the initial structures were sufficiently close to a minimum for all degrees of freedom included in the calculations. Vibrational frequencies were computed from the eigenvalues of the projected Hessian, $\widetilde{\mathcal{H}}^{sub}$, as follows

$$\mathbf{U}^T\,\widetilde{\mathcal{H}}^{sub}\,\mathbf{U} \,=\, \Lambda; \qquad \mathbf{v} = \frac{\sqrt{\Lambda}}{2\pi} \tag{1}$$

where Λ is the diagonal matrix of eigenvalues and $\mathbf{v} = (\nu_1, \nu_2, \ldots, \nu_{3N})$ is a vector containing the mode frequencies; for details see Ref. [1]. Six overall translational and rotational modes with a frequency of 0.0 cm^{-1} (from $1{\cdot}10^{-6}$ to $6{\cdot}10^{-2}$ cm^{-1}) were found for both structures. In

post-rigor, three additional modes with frequencies of 0.0 cm^{-1} (from $2 \cdot 10^{-7}$ to $3 \cdot 10^{-7}$ cm^{-1}) were also present; these modes are due to the spherical symmetry of the block containing the Mg^{+2} ion, i.e., the energy of the system does not change upon rotation of that block. Normal modes starting with mode 7 in rigor-like and with mode 10 in post-rigor describe the internal degrees of freedom of the myosin molecule in the two functional states. They were used to study how the intrinsic flexibility of the myosin molecule and its subdomains is exploited to make the rigor-like/post-rigor conformational transition.

We note that the BNM approach is exact for the model only in the harmonic approximation, i.e., in close proximity of the minima of the potential energy surface. Nevertheless, for hinge-like motions we expect that only a relatively small part of the protein will deviate from the harmonic model. Hence, in accord with the view that most of the structural plasticity of myosin arises from the hinges [6], we assume that large deformations of the system, such as the ones involved in the rigor-like/post-rigor conformational transition, can be captured by the NMSM path. Also, we note that although elastic energies (see below) computed along the NMSM path are very large (i.e., they have no physical meaning) local minimization of the actual structures in the full anharmonic potential shows that the transition path involves rather small energy changes (see Figure 1). Hence, we conclude that despite its limitations, the NMSM superposition model provides a useful representation of the transition path that can be refined (e.g., by a potential of mean force calculation [7]) to obtain a more accurate description of the energetics involved.

Normal Mode Analysis

Overlap Coefficients. To compare individual modes of the rigor-like and post-rigor conformations, the overlap between pairs of eigenvectors belonging to different functional states were computed (see "Main Text"). Normal mode overlaps are used to study the correlation of the eigenvectors in different structural states and to identify "robust" collective motions, which are likely to be functional [8]. In the case of myosin, where the various functional states are chemically not the same, a difficulty in the overlap analysis arises from the fact that the length of the eigenvectors differ. To compute the correlation map, the eigenvector components relative to the ligands (i.e., SO_4^{-2} ion in rigor-like, and

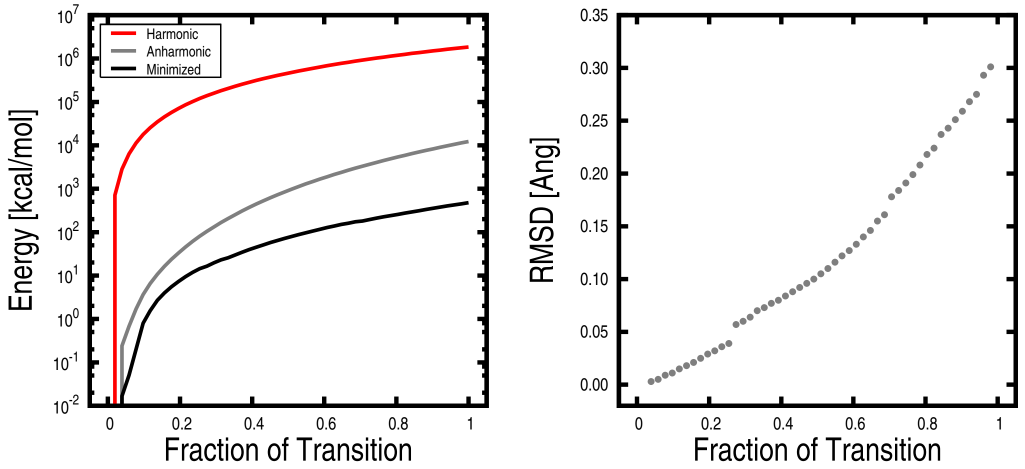

FIG. 1: Energetics along the rigor-like to NMSM post-rigor transition. On the left is shown the elastic harmonic energy and the CHARMM anharmonic energy before and after minimization are shown along the pathway. The applied minimization protocol consists of 10 cycles of 10000 steepest descent (SD) steps plus 10000 adopted basis Newton-Raphson (ABNR) steps with decreasing harmonic restraints on the backbone atoms; i.e., initial and final force constants of 1000 to 2 kcal/mol/Å^2 were applied, respectively. On the right is shown the all-atom RMSD between the original NMSM path and the structures resulting from energy minimization in the full anharmonic potential. Upon energy minimization, which results in very little structural changes (i.e., max RMSD $\sim$ 0.3 Å), the NMSM transition path energy is significantly reduced.

ATP and Mg^{+2} in post-rigor) were removed. The resulting vectors were re-orthogonalized by applying the Gram-Schmidt orthogonalization method [9] and then re-normalized prior to comparison. The overlap analysis was also repeated by simply projecting the ligands out and re-normalizing the resulting vectors. Although the resulting vectors are not orthogonal, given the small size of the ligands with respect to the myosin molecule (i.e., 5 and 38 atoms versus 8994 atoms for rigor-like and post-rigor, respectively), the low-frequency normal modes were not significantly altered by the ligand elimination and the same overlaps were obtained. In this study, the orthonormal basis set formed by the eigenvectors given by the Gram-Schmidt orthogonalization method were used for consistency and mathematical rigour.

Dynamic Domain Analysis. One possible way of identifying domains and hinge regions is to use the computational tool DynDom [10] (version 1.5) to analyze the interdomain conformational change described by the low-frequency modes; see Table I for a list of parameters used in the analysis. For each considered BNM vector, the two molecular structures corresponding to the extremes of the harmonic oscillation at a given temperature were compared. The analysis of the lowest-frequency modes shows a different dynamic behavior of the converter in the rigor-like and post-rigor states (see "Main Text").

DynDom Parameter	Used Value	Default Value
Window length (number of residues)	5	5
Minimum domain size (number of residues)	20	20
Minimum ratio of external to internal displacement	1.5	1.0

TABLE I: Dynamic Domain Analysis: set of parameters used in the DynDom runs; default values are reported for comparison. In the analysis of the rigor-like and post-rigor lowest-frequency modes, default parameters were used except for the minimum ratio of external to internal displacement, which is set to 1.5 to discourage excessive fragmentation.

[1] Li G, Cui Q (2002) A coarse-grained normal mode approach for macromolecules: An efficient implementation and application to Ca^{2+}-ATPase. Biophys J 83:2457–2474.

[2] Li G, Cui Q (2004) Analysis of functional motions in "Brownian molecular machines" with an efficient block normal mode approach. Biophys J 86:743–763.

[3] Lazaridis T, Karplus M (1999) Effective energy function for proteins in solution. Proteins: Structure, Function and Genetics 35:133–152.

[4] Tama F, Gadea FX, Marques O, Sanejouand Y (2000) Building-block approach for determining low-frequency normal modes of macromolecules. Proteins: Structure, Function and Genetics 41:1.

[5] Tirion MM (1996) Large amplitude elastic motions in proteins from a single-parameter, atomic analysis. Phys Rev Lett 77:1905–1908.

[6] Houdusse A, Sweeney H (2001) Myosin motors: missing structures and hidden springs. Curr Opin Struct Biol 11:182–194.

[7] Kirkwood JG (1935) Statistical mechanics of fluid mixtures. J Chem Phys 3:300–313.

[8] Nicolay S, Sanejouand YH (2006) Functional modes of proteins are among the most robust. Phys Rev Lett 96:078104.

[9] Schmidt E (1907) Zur theorie der linearen und nichtlinearen integralgleichungen. I. Teil: Entwicklung willkürlicher funktionen nach systemen vorgeschriebener. Math Ann 63:433–476.

[10] Hayward S, Berendsen HJC (1998) Systematic analysis of domain motions in proteins from conformational change. New results on citrate synthase and T4 Lysozyme. Proteins: Structure, Function and Genetics 30:144–154.
