# Supplementary material for: Allosteric Communication in Myosin V: From Small Conformational Changes to Large Directed Movements
Source: PLoS Comput Biol. 2008 Aug 15;4(8):e1000129. doi: 10.1371/journal.pcbi.1000129 (PMC2497441; doi:10.1371/journal.pcbi.1000129)
Supplement: Text S3 — Normal mode analysis of the rigor to apo post-rigor transition. (0.16 MB PDF) [file pcbi.1000129.s005.pdf]

Normal Mode Analysis of the Rigor to *apo* Post-rigor Transition (Text S3)

The *apo* post-rigor state was generated by removing Mg.ATP from a not fully relaxed post-rigor conformation (i.e., the coordinates of the backbone atoms were the same as in the X-ray post-rigor structure). The ligands were removed and the molecular structure energy minimized by applying the protocol described above (see Text S1). The effective energy and the gradient of the final *apo* post-rigor structure are -28751.5 kcal/mol and 0.0054 kcal/mol Å^2, respectively; see Table I in Text S1 for a comparison with the energy-minimized rigor and post-rigor structures. The *apo* post-rigor conformation does not differ very much from the post-rigor ATP-bound state (see Table I); the removal of Mg.ATP from the post-rigor structure followed by a deep energy minimization does not introduce significant conformational changes.

RMSD (Å)	apo-post/rigor (**a/r**)	apo-post/post-rigor (**a/p**)	rigor/post-rigor (**r/p**)
all-atom	5.42	0.54	5.40
C_α	5.14	0.50	5.20

TABLE I: Analysis of the rigor, post-rigor, and *apo* post-rigor structures used for the BNM calculations. The comparison between the *apo* post-rigor and post-rigor conformations shows that there is a small structural change.

Given the proximity of the central β-sheet to the myosin active site and the proposed role for ATP binding as the triggering event for its partial untwisting (see "Main Text"), the twist angle, τ, was computed for the three structures. As shown in Table II, τ values for the post-rigor and *apo* post-rigor structures are essentially the same and much smaller than the rigor value. Thus, the deletion of Mg.ATP and minimization does not promote a conformational transition of the β-sheet from partially untwisted to twisted.

τ (deg)	rigor (**r**)	post-rigor (**p**)	apo post (**a**)
C_α	108.7	94.1	93.3
backbone	106.0	91.6	90.4

TABLE II: β-sheet twist in the rigor, post-rigor, and *apo* post-rigor structures. Twist values are computed by fitting both the C_α and backbone atoms of the individual strands.

The energy-minimized *apo* post-rigor structure was used for the BNM calculation. No mode with a negative frequency was detected. The frequency of the 40 lowest-frequency

modes are reported in Table III; these values do not include the six zero-frequency modes corresponding to pure rotations and translations. The normal mode frequencies in *apo* post-

Mode #	**rigor** ω^R	**post** ω^P	**apo** ω^A	Mode #	**rigor** ω^R	**post** ω^P	**apo** ω^A
**1**	0.224	0.296	0.243	**21**	3.452	3.296	3.161
**2**	0.429	0.439	0.406	**22**	3.480	3.378	3.308
**3**	0.584	0.530	0.530	**23**	3.569	3.519	3.420
**4**	0.936	0.950	0.929	**24**	3.669	3.547	3.620
**5**	1.208	1.144	1.149	**25**	3.827	3.652	3.643
**6**	1.244	1.318	1.294	**26**	3.971	3.791	3.799
**7**	1.573	1.466	1.462	**27**	4.012	3.900	3.918
**8**	1.694	1.677	1.643	**28**	4.098	3.994	3.988
**9**	1.789	1.789	1.788	**29**	4.292	4.113	4.052
**10**	1.982	1.840	1.851	**30**	4.320	4.240	4.156
**11**	2.044	2.037	1.987	**31**	4.633	4.261	4.268
**12**	2.305	2.245	2.217	**32**	4.665	4.439	4.357
**13**	2.424	2.352	2.290	**33**	4.789	4.500	4.483
**14**	2.697	2.369	2.372	**34**	4.896	4.547	4.529
**15**	2.799	2.546	2.486	**35**	4.950	4.682	4.628
**16**	2.860	2.729	2.635	**36**	5.099	4.732	4.664
**17**	2.933	2.889	2.730	**37**	5.150	4.830	4.802
**18**	3.084	2.940	2.935	**38**	5.182	4.905	4.841
**19**	3.108	3.006	2.954	**39**	5.290	5.017	4.957
**20**	3.310	3.037	3.022	**40**	5.328	5.123	5.090

TABLE III: Normal mode frequencies of the 40 lowest-frequency modes in the rigor, post-rigor and *apo* post-rigor structures.

rigor are similar to the ones in post-rigor, though small deviations especially in the very low-frequency range are observed. The normal mode vectors of the *apo* post-rigor state were compared to the ones in rigor and post-rigor by computing the overlap coefficients (see "Main Text"). Figure 1 shows three overlap maps computed by considering the 40 lowest-frequency modes of each state. The post-rigor and *apo* post-rigor vectors are highly correlated in the entire range of frequencies. Moreover, the rigor/post-rigor and the rigor/*apo* post-rigor maps show very similar patterns. These results indicate that the effect of the removal of ATP on the normal mode vectors is essentially negligible.

Comparative involvement coefficients (IC) analyses performed on the rigor/post-rigor, post-rigor/rigor, and *apo* post-rigor/rigor transitions provide the last piece of information. Three cumulative profiles corresponding to the rigor/post-rigor (red), post-rigor/rigor (green), and *apo* post-rigor/rigor transitions are shown in Figure 2. The differences observed

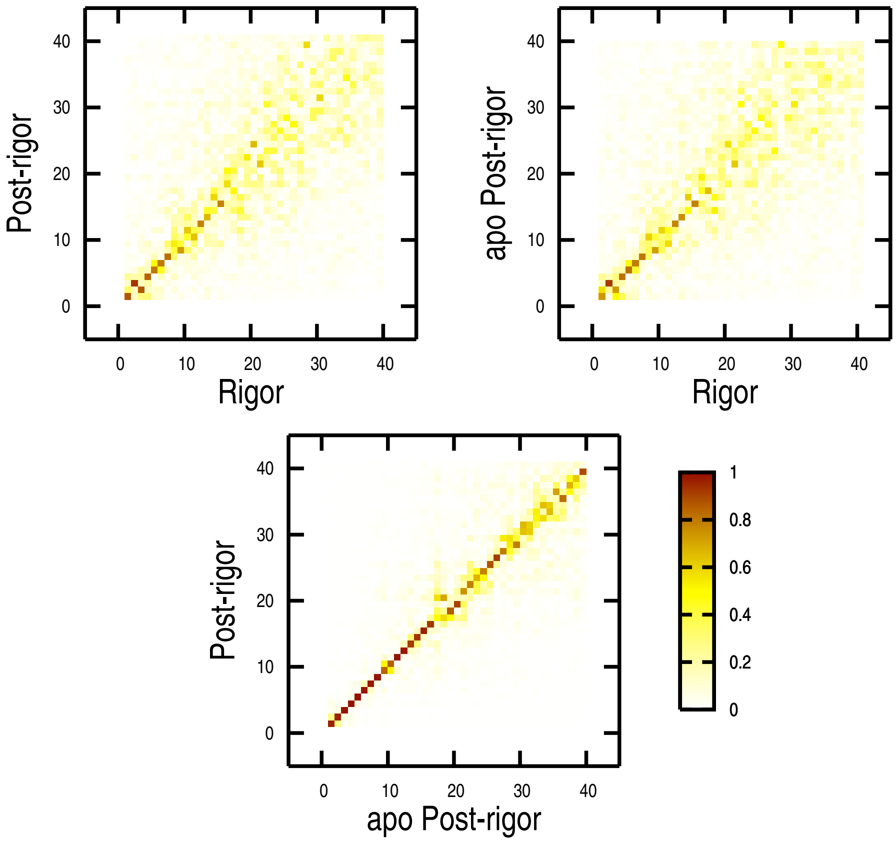

FIG. 1: The rigor/post-rigor, rigor/*apo*-post, and *apo*-post/post-rigor normal mode overlaps. Overlap values were computed for the first 40 lowest-frequency eigenvectors of each structure. The correlation between the mode vectors of the *apo* post-rigor and the post-rigor structure as well as the very similar patterns shown by the rigor/post-rigor and rigor/*apo*-post maps are striking.

in the normal mode activation pattern of the "forward" and the "backward" rigor/post-rigor transition are conserved in the absence of ATP; i.e., compare the green (with ATP) with the black (without ATP) profile.

The results of the normal mode analysis indicate that the physical presence of the nucleotide in the myosin active site is much less important than the difference in conformation between the rigor-like and post-rigor structures. The deletion of Mg.ATP from post-rigor slightly modifies the frequencies of the modes while leaving essentially unaltered the direction of the corresponding mode vectors. Hence, the primary effect of ATP is to stabilize the post-rigor structure, rather than to alter the intrinsic myosin flexibility. In this sense the effect is "indirect" because it promotes the transition to a different region of the conformational

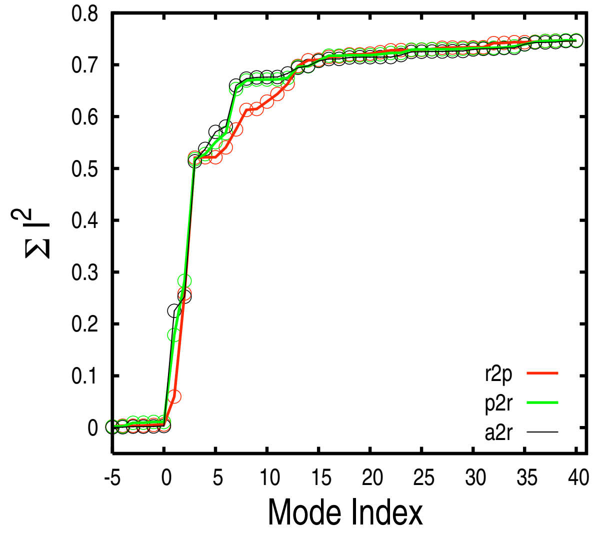

FIG. 2: Cumulative involvement-coefficient analyses for the rigor/post-rigor (in red), post-rigor/rigor (in green), and *apo* post-rigor/rigor transitions (in black).

space that is associated to an important change in the direction of the modes.
