# Supplementary material for: Allosteric Communication in Myosin V: From Small Conformational Changes to Large Directed Movements
Source: PLoS Comput Biol. 2008 Aug 15;4(8):e1000129. doi: 10.1371/journal.pcbi.1000129 (PMC2497441; doi:10.1371/journal.pcbi.1000129)
Supplement: Text S4 — Screw axis determination. (0.72 MB PDF) [file pcbi.1000129.s006.pdf]

Screw Axis Determination (Text S4)

Given two molecular conformations $\tilde{\mathbf{X}}_{\mathbf{A}}$ and $\tilde{\mathbf{X}}_{\mathbf{B}}$, whose position and orientation in the laboratory frame are defined respectively by the coordinates of their center of mass, $\tilde{\mathbf{r}}_A$ and $\tilde{\mathbf{r}}_B$, and three Euler angles, $\tilde{\boldsymbol{\Omega}}_A = (\alpha, \beta, \gamma)_A$ and $\tilde{\boldsymbol{\Omega}}_B = (\alpha, \beta, \gamma)_B$, any transformation that optimally superposes the former to the latter can be expressed as a linear combination of a translation and a rotation. The best-fit algorithm proposed by Kabsch [1] translates the starting conformation, $\tilde{\mathbf{X}}_{\mathbf{A}}$, to the center of mass of the target and finds the rotation matrix, $\mathcal{M}$, that optimally aligns the two conformations. From Euler's rotation theorem, any rotation can be defined by a single angle of rotation, φ, and the direction of a unit vector, $\hat{\mathbf{v}} = (x, y, z)$, about which to rotate.

$$\mathcal{M}(\hat{\mathbf{v}}, \varphi) = \begin{bmatrix} \cos\varphi + (1-\cos\varphi)\, x^2 & (1-\cos\varphi)\, xy - (\sin\varphi) z & (1-\cos\varphi)\, xz + (\sin\varphi)\, y \\ (1-\cos\varphi)\, yx + (\sin\varphi)\, z & \cos\varphi + (1-\cos\varphi)\, y^2 & (1-\cos\varphi)\, yz - (\sin\varphi)\, x \\ (1-\cos\varphi)\, zx - (\sin\varphi)\, y & (1-\cos\varphi)\, zy + (\sin\varphi) x & \cos\varphi + (1-\cos\varphi)\, z^2 \end{bmatrix} \quad (1)$$

Thus, for a given transformation, whose corresponding rotation matrix is known, $\hat{\mathbf{v}}$ and φ are determined by combining the non-diagonal elements of $\mathcal{M}$, so as to obtain

$$\vec{v} = 1/2 \begin{vmatrix} \mathcal{M}_{32} - \mathcal{M}_{23} \\ \mathcal{M}_{13} - \mathcal{M}_{31} \\ \mathcal{M}_{21} - \mathcal{M}_{12} \end{vmatrix} = \begin{vmatrix} (\sin\varphi)\, x \\ (\sin\varphi)\, y \\ (\sin\varphi)\, z \end{vmatrix} = (\sin\varphi)\, \hat{\mathbf{v}} \quad (2)$$

Since $\hat{\mathbf{v}}$ is a unit vector (i.e., $|\hat{\mathbf{v}}| = 1$), by computing the square of both sides of Eq. 2, the dependency from $\hat{\mathbf{v}}$ vanishes

$$\vec{v}^2 = (\sin^2\varphi)\ \hat{\mathbf{v}}^2 = \sin^2\varphi, \quad (3)$$

and φ and $\hat{\mathbf{v}}$ are obtained as

$$\varphi = \arcsin(|\vec{v}|) \quad (4)$$

$$\hat{\mathbf{v}} = (1/\sin\varphi)\ \vec{v} \quad (5)$$

The best-fit transformation can be described by a translation along $\tilde{\mathbf{r}}_{\mathrm{AB}} = \tilde{\mathbf{r}}_B - \tilde{\mathbf{r}}_A$, i.e., the vector joining the centers of mass of the two conformations, followed by a rotation around $\hat{\mathbf{v}}$ by the angle φ. The same transformation, including both translational and rotational contributions, can be expressed by means of a screw axis, $\mathcal{V}$, parallel to $\hat{\mathbf{v}}$ but positioned differently; i.e., the center of rotation, $\tilde{\mathbf{v}}_{\mathrm{c}}$, does not necessarily coincide with the center of

mass of the target. The advantage of using a screw axis is that the $\tilde{\mathbf{X}}_{\mathbf{A}} \rightarrow \tilde{\mathbf{X}}_{\mathbf{B}}$ translation of the center of mass can be, at least partially, described in terms of the rotation used to optimally align the two conformations; i.e., the rotation about $\mathcal{V}$ accounts for the displacement component that is orthogonal to the rotation axis. The overall transformation is then completed by a translation along $\mathcal{V}$ that is less than or equal to $|\tilde{\mathbf{r}}_{\mathrm{AB}}|$. To determine the position of $\mathcal{V}$, $\tilde{\mathbf{r}}_{\mathrm{AB}}$ is projected onto the plane orthogonal to $\hat{\mathbf{v}}$ and passing through $\tilde{\mathbf{r}}_{\mathrm{A}}$

$$\begin{aligned} \tilde{\mathbf{r}}'_{\mathrm{A}} &= \tilde{\mathbf{r}}_{\mathrm{A}} \\ \tilde{\mathbf{r}}'_{\mathrm{B}} &= \tilde{\mathbf{r}}_{\mathrm{B}} - \left|\tilde{\mathbf{r}}_{\mathrm{AB}} \cdot \hat{\mathbf{v}}\right| \cdot \hat{\mathbf{v}} \end{aligned} \tag{6}$$

with $\tilde{\mathbf{r}}'_{\mathrm{A}}$ and $\tilde{\mathbf{r}}'_{\mathrm{B}}$ corresponding to the projections of $\tilde{\mathbf{r}}_{\mathrm{A}}$ and $\tilde{\mathbf{r}}_{\mathrm{B}}$. With these definitions, $\tilde{\mathbf{r}}'_{\mathrm{AB}} = \tilde{\mathbf{r}}'_{B} - \tilde{\mathbf{r}}'_{A}$ is orthogonal to $\hat{\mathbf{v}}$ and the transformation mapping $\tilde{\mathbf{r}}'_{\mathrm{A}}$ in $\tilde{\mathbf{r}}'_{\mathrm{B}}$ can be described by a pure rotation about $\mathcal{V}$ (see Figure 1). Imposing the constraint that the amplitude of

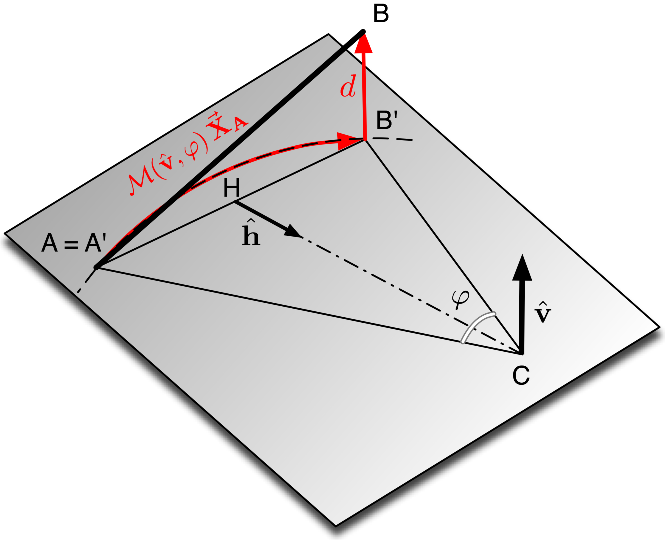

FIG. 1: Pictorial representation of the $\tilde{\mathbf{X}}_{\mathbf{A}} \rightarrow \tilde{\mathbf{X}}_{\mathbf{B}}$ screw-axis transformation. This geometric construction allows to express the position of the screw-axis, $\tilde{\mathbf{v}}_{\mathrm{c}}$, by simple trigonometric relationships involving the rotation angle φ (see text). The red arrows indicate the rotational, $\mathcal{M}(\hat{\mathbf{v}}, \varphi)\,\tilde{\mathbf{X}}_{\mathbf{A}}$, and translational, d, components of the overall transformation.

the rotation is equal to φ makes it possible to determine $\tilde{\mathbf{v}}_{\mathrm{c}}$ uniquely by simple trigonometric

relationships

$$\begin{aligned}\overline{\mathrm{HC}} &= \overline{\mathrm{AH}} \cdot \cot(\varphi/2) \\ \overline{\mathrm{AH}} &= \left|\tilde{\mathbf{r}}'_{\mathrm{AB}}\right|/2 \end{aligned} \tag{7}$$

As shown by Figure 1, the position of the screw axis $\mathcal{V}$ can be expressed as

$$\tilde{\mathbf{v}}_{\mathrm{C}} = \tilde{\mathbf{r}}_{\mathrm{H}} + \overline{\mathrm{HC}} \cdot \hat{\mathbf{h}} \tag{8}$$

with $\tilde{\mathbf{r}}_{\mathrm{H}}$ and $\hat{\mathbf{h}}$ defined as

$$\tilde{\mathbf{r}}'_{\mathrm{H}} = \frac{\tilde{\mathbf{r}}'_{\mathrm{A}} + \tilde{\mathbf{r}}'_{\mathrm{B}}}{2} \tag{9}$$

$$\hat{\mathbf{h}} = \hat{\mathbf{v}} \times \frac{\tilde{\mathbf{r}}'_{\mathrm{AB}}}{|\tilde{\mathbf{r}}'_{\mathrm{AB}}|} \tag{10}$$

By substituting Eqs. 7, 9 and 10 into Eq. 8, one obtains

$$\tilde{\mathbf{v}}_{\mathrm{c}} = \frac{1}{2}\left[\tilde{\mathbf{r}}'_{\mathrm{A}} + \tilde{\mathbf{r}}'_{\mathrm{B}} + \cot(\varphi/2)\,(\hat{\mathbf{v}} \times \tilde{\mathbf{r}}'_{\mathrm{AB}})\right] \tag{11}$$

To complete the screw-axis transformation, the translational contribution along the direction of $\hat{\mathbf{v}}$ is given by

$$\tilde{\mathbf{d}} = \left|\tilde{\mathbf{r}}_{\mathrm{AB}} \cdot \hat{\mathbf{v}}\right| \hat{\mathbf{v}} \tag{12}$$

with the modulus of $\tilde{\mathbf{d}}$, $|\tilde{\mathbf{d}}| = \left|\tilde{\mathbf{r}}_{\mathrm{AB}} \cdot \hat{\mathbf{v}}\right|$, referred to as the screw-axis translational shift, d. Overall, the $\tilde{\mathbf{X}}_{\mathbf{A}} \rightarrow \tilde{\mathbf{X}}_{\mathbf{B}}$ screw-axis transformation is defined as

$$\tilde{\mathbf{X}}_{\mathbf{B}} = \left[\mathcal{M}(\hat{\mathbf{v}}, \varphi)\left(\tilde{\mathbf{X}}_{\mathbf{A}} - \tilde{\mathbf{v}}_{\mathrm{c}}\right) + \tilde{\mathbf{v}}_{\mathrm{c}}\right] + d\hat{\mathbf{v}} \tag{13}$$

which is a linear combination of a rotation and a translation (see red arrows in Figure 1). As shown by Eqs. 12 and 13, if $\tilde{\mathbf{r}}_{\mathrm{AB}}$ and $\hat{\mathbf{v}}$ are orthogonal the translational component vanishes. By contrast, if $\tilde{\mathbf{r}}_{\mathrm{AB}}$ and $\hat{\mathbf{v}}$ are parallel, d is equal to the distance between the centers of mass of the two conformations and the translational shift is equal to $|\tilde{\mathbf{r}}_{\mathrm{AB}}|$; i.e., it coincides with the one given by Kabsch's algorithm. Thus, Eq. 13 shows that the introduction of a screw axis $\mathcal{V}$ minimizes the translational contribution to the overall transformation in favor of the rotational contribution. The screw-axis transformation, which we use in the analysis to describe the rigor-like/post-rigor transition of the myosin subdomains minimizes the translational contributions (cfr. the individual subdomain rigor/post-rigor center-of-mass distances and the screw axes translational shifts in Table III in "Main Text") and provides

a more accurate description of the large-amplitude motions along the NMSM pathway. In fact, this representation uses the original alignment of the crystal structures, which is based on the optimal superposition of all C_α atoms, and removes the bias introduced by the choice of the alignment.

Rigor-like to NMSM Post-rigor Screw-axis Transformation

The overall rigid-body motion of the myosin subdomains along the NMSM transition is best described in terms of individual screw axes (see "Main Text"). The screw axes defined in Table III were obtained from the knowledge of the rigor-like and NMSM post-rigor structures by following the procedure described above; where $\tilde{\mathbf{X}}_{\mathbf{A}}$ and $\tilde{\mathbf{X}}_{\mathbf{B}}$ correspond to the rigor-like and NMSM post-rigor conformations upon their optimal superposition. Given the initial ($\tilde{\mathbf{X}}^i_{\mathbf{A}}$) and final ($\tilde{\mathbf{X}}^i_{\mathbf{B}}$) conformations of the i-th subdomain, the rotational angle, φ^i, the orientation, $\hat{\mathbf{v}}^i$, the position, $\tilde{\mathbf{v}}^i_{\mathrm{c}}$, and the translational shift d^i of the corresponding screw axis were determined by solving Eqs. 4, 5, 11 and 12; these four quantities univocally define the screw axis. The resulting screw axes were used to generate an NMSM post-rigor structure by pure rigid-body motions. For this purpose, the transformation described by Eq. 13 was applied to each subdomain by starting from its conformation in the rigor-like state ($\tilde{\mathbf{X}}^i_{\mathbf{A}}$). The resulting "rigid-body" post-rigor conformation was used to elucidate the origin of the partial untwisting of the β-sheet in the rigor to post-rigor transition of myosin (see "Main Text"). We note that the "rigid-body" post-rigor structure differs from the NMSM post-rigor conformation, despite the fact that the former has been obtained using the latter. The conformational difference is due to the more "plastic" character of the NMSM transition which, unlike the "rigid-body" transformation, allows the relaxation of the structure along the pathway.

[1] Kabsch W (1976) A solution for the best rotation to relate two sets of vectors. Acta Cryst A32:922–923.
