# Supplementary material for: Allosteric Communication in Myosin V: From Small Conformational Changes to Large Directed Movements
Source: PLoS Comput Biol. 2008 Aug 15;4(8):e1000129. doi: 10.1371/journal.pcbi.1000129 (PMC2497441; doi:10.1371/journal.pcbi.1000129)
Supplement: Text S5 — Flexibility of the subdomain linkers. (0.07 MB PDF) [file pcbi.1000129.s007.pdf]

Flexibility of the Subdomain Linkers (Text S5)

It is useful to distinguish between the inherent "flexibility" of the myosin subdomains, secondary structure elements, and linkers and their "mobility". As defined here, the inherent flexibility, which implies a change in shape either in the observed transition or during the normal mode oscillations, is obtained by computing the RMS deviation after optimal superposition of the C_α atoms of the particular structural element; the mobility is calculated correspondingly after optimal superposition of the entire molecule (and includes the smaller contribution from the flexibility). In the former case, the calculated value corresponds to the deformation of the selected region during the transition; in the latter, it reflects the amplitude of the displacement independently of the change in shape. Since, this type of analysis can be applied to any pairs of structures, the comparison of the molecular conformations corresponding to the maximal amplitude of the normal mode oscillations with the equilibrium structure provides information on the *dynamic* flexibility of any given structural element inherent in the particular myosin state.

The *dynamic* flexibility of the various subdomain linkers, i.e., the P-loop, switch I, switch II, the strut, and helix SH1, in the rigor-like and post-rigor states was analyzed. For this purpose, flexibility and mobility values were computed for the 40 lowest-frequency modes at 300 K and averaged. As can be seen in Table I, mobility values are much larger than flexibility values for all subdomain linkers. The flexibility of the P-loop, switch I, and switch

**Subdomain Linkers**	Number of Residues	**rigor-like**			**post-rigor**		
		Flexibility	Mobility	Mob/Flex	Flexibility	Mobility	Mob/Flex
P-loop	9	0.004	0.032	8.0	0.003	0.028	9.3
switch I	11	0.005	0.030	6.0	0.005	0.031	6.2
switch II	12	0.009	0.035	3.9	0.009	0.034	3.7
strut	6	0.008	0.041	5.1	0.010	0.044	4.4
SH1 helix	10	0.006	0.036	6.0	0.006	0.045	7.5

TABLE I: Mobility and flexibility of various subdomain linkers in the myosin head as described by the 40 lowest-frequency modes of the rigor-like and post-rigor states. Mobility and flexibility values are given in Å.

II linkers are compared in Figure 1. P-loop and switch I have similar flexibility values, which correspond to about half that of switch II. Moreover, the *dynamic* flexibility of the ensemble of the three nucleotide-binding elements, which is referred to as the "ATP site" in Figure 1,

is 4.8, 3.0, and 1.6 times larger than the values computed for the P-loop, switch I, and switch II.

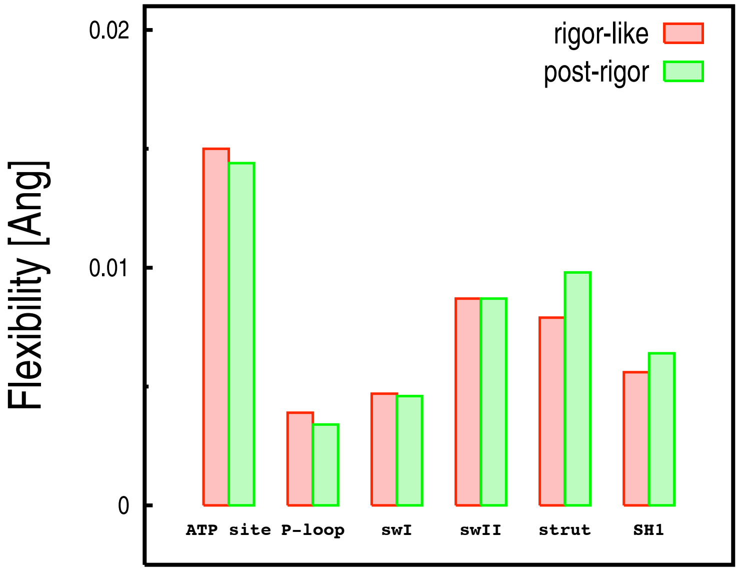

FIG. 1: Flexibility of the myosin subdomain linkers as observed from the rigor-like and post-rigor X-ray structures. Mobility and flexibility values are given in Å.

These results indicate a strong rigid-body character in the dynamics of the myosin subdomain linkers as described by the low-frequency normal modes. In particular, P-loop and switch I appear as the most rigid elements; they show the largest mobility/flexibility ratios (see Table I). In this scenario, conformational changes in the ATP binding site are likely to be described by combinations of rigid-body rearrangements of the nucleotide-binding elements, which are tightly coupled to the motion of the corresponding subdomains.
