# Supplementary material for: Allosteric Communication in Myosin V: From Small Conformational Changes to Large Directed Movements
Source: PLoS Comput Biol. 2008 Aug 15;4(8):e1000129. doi: 10.1371/journal.pcbi.1000129 (PMC2497441; doi:10.1371/journal.pcbi.1000129)
Supplement: Table S1 — Myosin V subdomains and linkers. (0.03 MB PDF) [file pcbi.1000129.s008.pdf]

**Subdomains**	Number of Residues	Amino Acids Involved
SH3	60	1-60
N	154	61-184 & 654-683
U50	230	199-340 & 354-438 & 575-593
L50	135	448-540 & 547-569 & 635-653
C	63	700-762
IQ	33	763-795
ELC	151	1-151
**Linkers**	**Number of Residues**	**Amino Acids Involved**
P-loop	9	161-169
switch I	11	209-219
switch II	12	437-448
strut	6	569-574
SH1 helix	10	687-696

TABLE S1: The myosin V subdomains and linkers. For each element the name, the number of residues, the amino acids involved are specified.
