# Supplementary material for: Allosteric Communication in Myosin V: From Small Conformational Changes to Large Directed Movements
Source: PLoS Comput Biol. 2008 Aug 15;4(8):e1000129. doi: 10.1371/journal.pcbi.1000129 (PMC2497441; doi:10.1371/journal.pcbi.1000129)
Supplement: Table S2 — Myosin V secondary-structure elements. (0.04 MB PDF) [file pcbi.1000129.s009.pdf]

**Secondary-Structure Elements**	Secondary Structure	Number of Residues	Amino Acids Involved	Protein Subdomain
HB	α-helix	15	G82-S96	N
HE	α-helix	15	I139-D153	N
HF	α-helix	15	K169-V183	N
HH	α-helix	15	V192-I206	U50
HI	α-helix	8	H260-A267	U50
HK	α-helix	14	D301-L314	U50
HL	α-helix	18	D319-G336	U50
HM	α-helix	10	D355-M364	U50
HN	α-helix	8	Y368-L375	U50
HO	α-helix	31	K393-L423	U50
HP	α-helix	18	F449-N466	L50
Relay	α-helix	14	M467-E480	L50
HP-Relay	α-helix	32	F449-E480	L50
HQ	α-helix	8	Q493-E500	L50
HR	α-helix	10	V505-K514	L50
HS	α-helix	12	D520-D531	L50
HT	α-helix	8	G563-V570	L50
HU	α-helix	9	E574-I582	U50
HW	α-helix	17	V636-A652	L50
SH1	α-helix	10	V687-G696	$\times$
SH2	α-helix	10	E675-L684	N
HX	α-helix	7	Q705-D711	C
HY	α-helix	12	R724-L735	C
HZ	α-helix	39	G754-R792	C/IQ
β_1	β-strand	4	Y100-C103	N
β_2	β-strand	5	V106-I110	N
β_3	β-strand	8	I655-S662	N
β_4	β-strand	6	Q157-S162	N
β_5	β-strand	8	S431-I438	U50
β_6	β-strand	8	G221-F228	U50
β_7	β-strand	10	I234-L243	U50
β_{sh}	β-sheet	49	β_1-β_7	$\times$

TABLE S2: The myosin V secondary-structure elements. For each element the name, the type of secondary structure, the number of residues, the amino acids involved, and the subdomain to which it belongs are specified.
