# Supplementary material for: Allosteric Communication in Myosin V: From Small Conformational Changes to Large Directed Movements
Source: PLoS Comput Biol. 2008 Aug 15;4(8):e1000129. doi: 10.1371/journal.pcbi.1000129 (PMC2497441; doi:10.1371/journal.pcbi.1000129)
Supplement: Table S3 — Rigor-like and post-rigor normal mode overlaps. (0.03 MB PDF) [file pcbi.1000129.s010.pdf]

rigor-like		post-rigor		overlap
Mode #	ω^R	Mode #	ω^P	$(L_i^R \cdot L_j^P)$
**1**	0.224	**1**	0.296	0.87
**2**	0.429	**3**	0.530	0.93
**3**	0.584	**2**	0.439	0.86
**4**	0.936	**4**	0.950	0.84
**5**	1.208	**5**	1.144	0.80
**6**	1.244	**6**	1.318	0.80
**7**	1.573	**7**	1.466	0.82
**9**	1.789	**8**	1.677	0.73
**10**	1.982	**11**	2.037	0.62
**11**	2.044	**10**	1.840	0.64
**12**	2.305	**12**	2.245	0.78
**13**	2.424	**13**	2.352	0.66
**15**	2.799	**15**	2.546	0.78
**20**	3.310	**24**	3.519	0.66
**21**	3.452	**21**	3.296	0.63

TABLE S3: Normal mode frequencies of highly correlated modes in the rigor-like and post-rigor states of myosin V. An overlap value of 0.6 was used as a lower-bound threshold in selecting highly correlated pairs. The ω values are given in wave numbers (cm^{-1}).
