# Supplementary material for: Allosteric Communication in Myosin V: From Small Conformational Changes to Large Directed Movements
Source: PLoS Comput Biol. 2008 Aug 15;4(8):e1000129. doi: 10.1371/journal.pcbi.1000129 (PMC2497441; doi:10.1371/journal.pcbi.1000129)
Supplement: Table S4 — Rigor-like and post-rigor normal mode frequencies. (0.03 MB PDF) [file pcbi.1000129.s011.pdf]

Mode	**rigor**	**post**	Mode	**rigor**	**post**
#	ω^R	ω^P	#	ω^R	ω^P
**1**	0.224	0.296	**21**	3.452	3.296
**2**	0.429	0.439	**22**	3.480	3.378
**3**	0.584	0.530	**23**	3.569	3.519
**4**	0.936	0.950	**24**	3.669	3.547
**5**	1.208	1.144	**25**	3.827	3.652
**6**	1.244	1.318	**26**	3.971	3.791
**7**	1.573	1.466	**27**	4.012	3.900
**8**	1.694	1.677	**28**	4.098	3.994
**9**	1.789	1.789	**29**	4.292	4.113
**10**	1.982	1.840	**30**	4.320	4.240
**11**	2.044	2.037	**31**	4.633	4.261
**12**	2.305	2.245	**32**	4.665	4.439
**13**	2.424	2.352	**33**	4.789	4.500
**14**	2.697	2.369	**34**	4.896	4.547
**15**	2.799	2.546	**35**	4.950	4.682
**16**	2.860	2.729	**36**	5.099	4.732
**17**	2.933	2.889	**37**	5.150	4.830
**18**	3.084	2.940	**38**	5.182	4.905
**19**	3.108	3.006	**39**	5.290	5.017
**20**	3.310	3.037	**40**	5.328	5.123

TABLE S4: Normal mode frequencies of the first 40 lowest-frequency modes of the rigor-like and post-rigor states of myosin V.
