# Supplementary material for: Allosteric Communication in Myosin V: From Small Conformational Changes to Large Directed Movements
Source: PLoS Comput Biol. 2008 Aug 15;4(8):e1000129. doi: 10.1371/journal.pcbi.1000129 (PMC2497441; doi:10.1371/journal.pcbi.1000129)
Supplement: Table S5 — Involvement coefficients specialized for the rigor-like/post-rigor transition of the motor domain and individual subdomains. (0.03 MB PDF) [file pcbi.1000129.s012.pdf]

Mode #	**Motor**		**N**		**U50**		**L50**		**C**	
	rigor	post	rigor	post	rigor	post	rigor	post	rigor	post
1	0.00	0.04	0.04	0.06	**-0.12**	0.05	0.09	-0.03	0.05	-0.01
2	0.00	0.08	-0.03	**-0.21**	-0.03	**0.18**	0.00	**-0.15**	0.08	-0.10
3	0.02	0.00	**-0.15**	-0.07	**0.14**	0.00	**-0.12**	0.01	0.01	0.08
4	0.02	0.03	0.07	-0.04	-0.03	-0.01	-0.02	0.05	-0.08	0.11
5	0.06	0.05	**-0.12**	**-0.14**	-0.01	0.08	**0.24**	**0.16**	-0.02	-0.07
6	0.07	0.06	0.02	0.03	**-0.21**	**0.22**	0.06	**-0.14**	0.10	-0.04
7	**0.12**	**0.28**	**-0.12**	**0.13**	**-0.13**	**0.21**	0.02	0.08	0.07	0.04
8	**0.20**	**0.35**	-0.06	**0.15**	-0.06	**0.21**	**-0.16**	**0.25**	**-0.15**	0.06
9	**0.18**	0.03	**0.12**	-0.03	**0.17**	-0.09	0.08	0.01	-0.02	0.11
10	**0.23**	0.06	0.05	-0.08	**0.23**	-0.04	**0.17**	0.10	-0.08	-0.10
11	**0.15**	0.09	-0.06	0.09	**-0.13**	-0.05	0.04	0.06	**-0.17**	0.07
12	**0.19**	0.09	**0.14**	-0.04	0.06	-0.07	**0.15**	-0.08	-0.02	0.06
13	**0.31**	**0.33**	**0.29**	**-0.19**	**0.13**	**-0.20**	**0.14**	**-0.20**	0.01	0.02
14	**0.20**	0.08	**-0.12**	0.00	0.01	-0.04	**-0.18**	0.04	-0.10	**-0.14**
15	**0.21**	**0.23**	-0.07	0.05	**-0.24**	**0.25**	0.02	-0.04	-0.09	**0.16**
16	**0.11**	**0.11**	**-0.16**	**0.12**	**0.14**	-0.10	**0.12**	0.11	0.08	**0.12**
17	0.09	0.02	**-0.20**	-0.04	0.02	0.01	**0.14**	**0.13**	**-0.14**	-0.09
18	**0.15**	0.07	**-0.20**	**-0.26**	-0.07	0.01	**0.12**	**0.15**	-0.09	-0.03
19	0.02	0.10	0.09	-0.02	**-0.12**	0.00	-0.05	-0.01	0.05	**-0.16**
20	0.00	0.02	**0.15**	**-0.14**	-0.07	0.04	-0.07	0.03	-0.01	-0.07
21	**0.17**	**0.15**	-0.04	**0.15**	0.08	-0.11	**0.15**	**-0.29**	**0.19**	-0.09
22	0.04	0.08	-0.08	-0.09	-0.05	0.02	-0.02	0.02	**0.14**	-0.08
23	**0.11**	0.01	-0.05	-0.01	0.06	0.01	**0.19**	**0.15**	0.02	**-0.22**
24	0.04	**0.17**	-0.05	**-0.17**	-0.03	-0.03	**0.13**	-0.03	-0.09	-0.09

TABLE S5: Rigor-like and post-rigor involvement coefficients corresponding to the conformational transition of the motor domain (including N, U50, L50, and C; aa 61-762), and individual subdomains N, U50, L50, and C, respectively. Modes that contribute more than 0.10 and 0.12 to the structural transition of the motor domain and its subdomains, respectively, are shown in bold. The values chosen as thresholds in the involvement-coefficient analysis are somewhat arbitrary but make possible the identification of modes that mostly contribute to specific conformational changes.
