# Supplementary material for: Allosteric Communication in Myosin V: From Small Conformational Changes to Large Directed Movements
Source: PLoS Comput Biol. 2008 Aug 15;4(8):e1000129. doi: 10.1371/journal.pcbi.1000129 (PMC2497441; doi:10.1371/journal.pcbi.1000129)
Supplement: Table S6 — Inter-subdomain center of mass distances from the rigor-like to the post-rigor and NMSM structures. (0.03 MB PDF) [file pcbi.1000129.s013.pdf]

Subdomains	**Rigor-like**	**NMSM**	**Post-rigor**	$\Delta_{\mathbf{M/R}}$	$\Delta_{\mathbf{P/R}}$
**N/U50**	36.0	36.2	36.0	0.2	0.0
**N/L50**	27.5	27.7	27.5	0.2	0.0
**U50/L50**	29.5	31.4	31.4	1.9	1.9
**N/C**	30.9	34.6	34.9	3.7	4.0
**U50/C**	64.1	67.7	67.9	3.6	3.8
**L50/C**	42.6	44.5	44.4	1.9	1.8

TABLE S6: Inter-subdomain center of mass distances; $\Delta_{\mathbf{M}}$ shows the distance obtained from the NMSM path, while $\Delta_{\mathbf{P}}$ the one from the post-rigor X-ray structure.
