# Supplementary material for: Allosteric Communication in Myosin V: From Small Conformational Changes to Large Directed Movements
Source: PLoS Comput Biol. 2008 Aug 15;4(8):e1000129. doi: 10.1371/journal.pcbi.1000129 (PMC2497441; doi:10.1371/journal.pcbi.1000129)
Supplement: Table S7 — Rigor-like/NMSM Δτ values between pairs of neighboring strands of the central β-sheet. (0.03 MB PDF) [file pcbi.1000129.s014.pdf]

β-strand pair	NMSM $\Delta\tau$ [deg]
$\beta_1\beta_2$	4.0
$\beta_2\beta_3$	2.9
$\beta_3\beta_4$	1.8
$\beta_4\beta_5$	2.3
$\beta_5\beta_6$	1.8
$\beta_6\beta_7$	1.1

TABLE S7: $\Delta\tau$ values between pairs of neighboring strands at the end of the NMSM path ($\Delta\tau_{ij} = \tau_{ij}(\text{rigor}) - \tau_{\text{ij}}(\text{NMSM})$).
