# Supplementary material for: Allosteric Communication in Myosin V: From Small Conformational Changes to Large Directed Movements
Source: PLoS Comput Biol. 2008 Aug 15;4(8):e1000129. doi: 10.1371/journal.pcbi.1000129 (PMC2497441; doi:10.1371/journal.pcbi.1000129)
Supplement: Table S8 — Flexibility of the converter β-sheet. (0.04 MB PDF) [file pcbi.1000129.s015.pdf]

τ_{ij} [deg]	β-strand pair	**rigor-like** min	**post-rigor** NMSM	**post-rigor** min
τ_{12}	$\beta_1\beta_2$	26.0	26.2	25.9
τ_{23}	$\beta_2\beta_3$	10.5	11.5	9.8
τ_{13}	$\beta_1\beta_3$	36.5	37.8	35.6
$\Delta\tau$		0.0	1.3	0.8

TABLE S8: Twist values for pairs of neighboring strands in the converter β-sheet. The values were computed for the energy-minimized rigor-like and post-rigor structures, and the NMSM post-rigor conformation. $\Delta\tau$ values relative to the rigor-like ($\Delta\tau = |\tau_{13}(\text{rigor}) - \tau_{13}(\text{X})|$) show that the three-stranded β-sheet is fairly rigid, so that it allows for efficient structural communication between the N and L50 subdomains.
