# Supplementary figures and images for: Allosteric Communication in Myosin V: From Small Conformational Changes to Large Directed Movements
Source: PLoS Comput Biol. 2008 Aug 15;4(8):e1000129. doi: 10.1371/journal.pcbi.1000129 (PMC2497441; doi:10.1371/journal.pcbi.1000129)

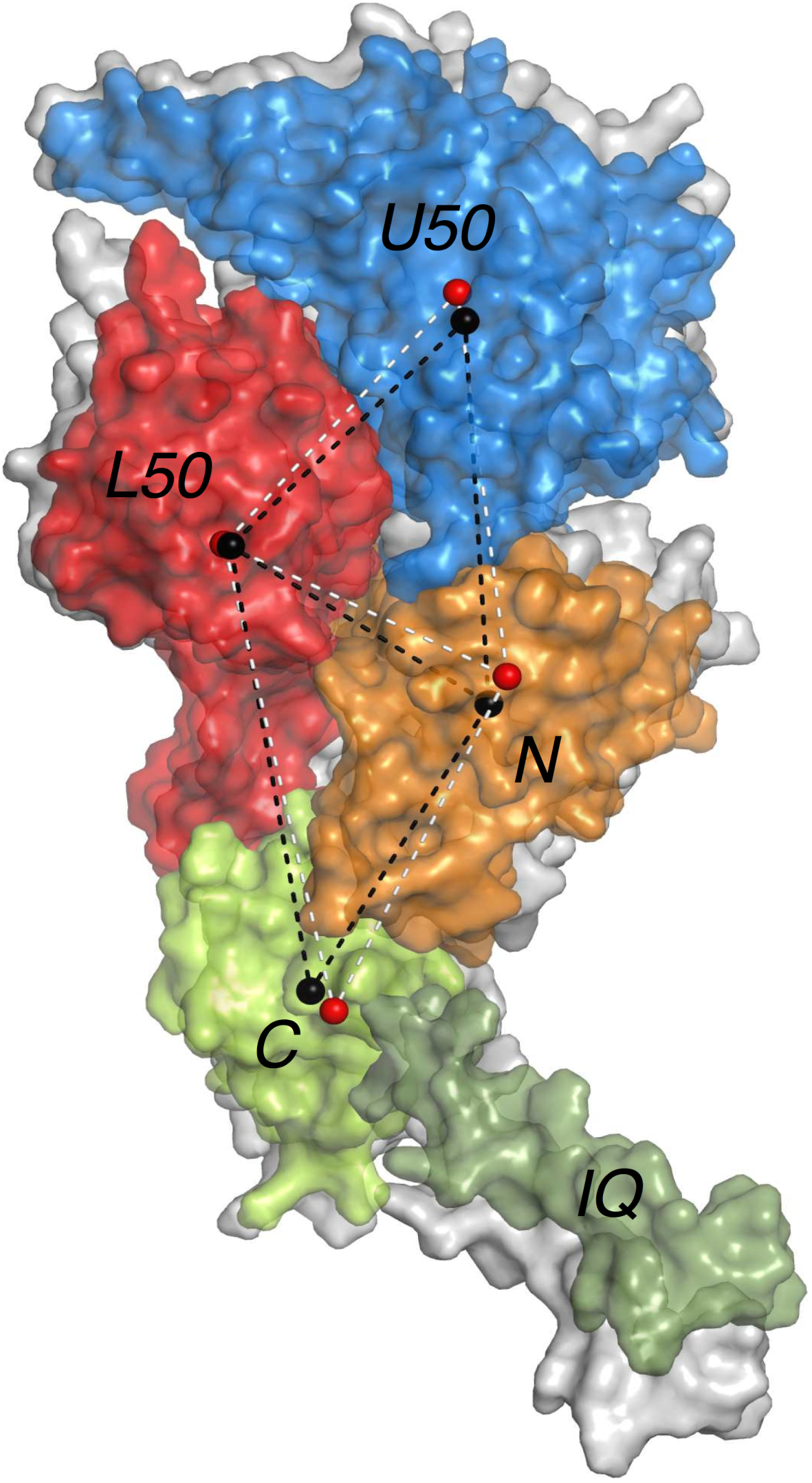

Supplement: Figure S1 — Center-of-mass displacement of the motor subdomains along the rigor to NMSM post-rigor transition. The rigor structure is shown in colors, the NMSM post-rigor conformation in grey. Black and red spheres indicate the position of the subdomains centers of mass in the rigor-like and the NMSM post-rigor conformation, respectively. The downward translation of converter (C) in the opposite direction to the translational motion of both N and U50 weakens the coupling between the neck region and the head domain. (2.44 MB TIF) [file pcbi.1000129.s016.tif]
